# Genus *Acanthella*—A Wealthy Treasure: Secondary Metabolites, Synthesis, Biosynthesis, and Bioactivities

**DOI:** 10.3390/md21040257

**Published:** 2023-04-21

**Authors:** Sabrin R. M. Ibrahim, Kholoud F. Ghazawi, Samar F. Miski, Duaa Fahad ALsiyud, Shaimaa G. A. Mohamed, Gamal A. Mohamed

**Affiliations:** 1 Preparatory Year Program, Department of Chemistry, Batterjee Medical College, Jeddah 21442, Saudi Arabia; 2 Department of Pharmacognosy, Faculty of Pharmacy, Assiut University, Assiut 71526, Egypt; 3 Clinical Pharmacy Department, College of Pharmacy, Umm Al-Qura University, Makkah 24382, Saudi Arabia; kfghazawi@uqu.edu.sa; 4 Department of Pharmacology and Toxicology, College of Pharmacy, Taibah University, Al-Madinah Al-Munawwarah 30078, Saudi Arabia; smiski@taibahu.edu.sa; 5 Department of Medical Laboratories-Hematology, King Fahd Armed Forces Hospital, Corniche Road, Andalus, Jeddah 23311, Saudi Arabia; duaaalsiyud@yahoo.com; 6 Faculty of Dentistry, British University, Suez Desert Road, Cairo 11837, Egypt; 7 Department of Natural Products and Alternative Medicine, Faculty of Pharmacy, King Abdulaziz University, Jeddah 21589, Saudi Arabia

**Keywords:** marine sponges, *Acanthella*, Axinellidae, nitrogen-containing terpenoids, life below water, health and wellbeing

## Abstract

Marine sponges are multicellular and primitive animals that potentially represent a wealthy source of novel drugs. The genus *Acanthella* (family Axinellidae) is renowned to produce various metabolites with various structural characteristics and bioactivities, including nitrogen-containing terpenoids, alkaloids, and sterols. The current work provides an up-to-date literature survey and comprehensive insight into the reported metabolites from the members of this genus, as well as their sources, biosynthesis, syntheses, and biological activities whenever available. In the current work, 226 metabolites have been discussed based on published data from the period from 1974 to the beginning of 2023 with 90 references.

## 1. Introduction

Natural metabolites from various sources, including microbes, animals, minerals, and plants, have been traditionally utilized for treating various human illnesses [1,2,3,4,5,6]. Recently, the developments in high-throughput screening and spectroscopic and analytical technologies have significantly boosted natural drug discovery, including marine-based drugs [7]. The marine environment is a rich source of a vast group of structurally unparalleled metabolites with diverse pharmacological activities that are reported from different marine organisms such as tunicates, sponges, mollusks, and bryozoans [8]. These metabolites are potential candidates for biotechnological applications and a number of them are in clinical trials; therefore, their impact on the pharmaceutical industry is continually growing [9,10,11]. They have been found to display an array of bioactivities, primarily antimicrobial, immunosuppressive, antifouling, anticancer, anthelmintic, antiprotozoal, neuroprotection, antiviral, and anti-inflammatory [7,8]. Among the marine organisms that have been investigated, sponges (Porifera), which are soft-bodied and sessile organisms, have become the focal point of natural product investigations due to the vast range of structurally unique biometabolites separated from these organisms [12,13].

The Axinellidae family (class Demospongiae, order Bubarida, family Dictyonellidae) [14] members, including *Axinella* and *Acanthella* genera, produce structurally varied terpenes with various nitrogen-containing functionalities [15]. Marine sponges of the genus *Acanthella* (Schmidt, 1862) are present in large numbers in the world’s oceans, especially in the South China Sea [16]. The accepted species of this genus and their status are listed in Table 1.

These sponges have been proven to be a prosperous source of sesquiterpenes and diterpenes featuring various nitrogen-containing groups, such as –NC (isonitrile), –NCS (isothiocyanate), –NCO (isocyanate), and –NHCHO (formamide) functionalities. Additionally, alkaloids and sterols are reportedly derived from these sponges. Some of these metabolites possess bioactivities such as antimalarial, cytotoxic, antimicrobial, anthelmintic, and antifouling [17,18,19,20,21,22,23,24,25]. Furthermore, some of them with promising bioactivities and unique structures have drawn the attention of chemists to investigate their synthesis in alignment with an in-depth biological evaluation for discovering new drug leads [26,27,28,29]. It is noteworthy that there are no available reported works that focus on the genus *Acanthella*. Therefore, this work aimed at highlighting the potential of this genus in terms of natural metabolite production. Herein, all reported studies on this genus are discussed, including secondary metabolites and their sources, as well as their biosynthesis, synthesis, and bioactivities whenever applicable.

## 2. Methodology

The reported studies on the genus *Acanthella* were collected by carrying out a literature search on different Publishers and databases, including Web of Science, PubMed, Scopus, Google-Scholar, Science Direct, Thieme, Bentham, Springer, Willey, and Taylor/Francis using the keywords: “*Acanthella* + compound” OR “*Acanthella* + NMR” OR “*Acanthella* + Biological activity” OR “*Acanthella* + nanoparticles” OR “*Acanthella* + Terpenoids” OR “*Acanthella* + Semi-synthesis”. This review involved investigations published in the English language in peer-reviewed journals in the period from 1974 to the beginning of 2023. A total of 85 published articles have been highlighted. The suggests irrelevant and non-reviewed journals’ published articles, as well as all non-English written articles, were not included. For the non-English work, the data were extracted from the English abstracts whenever available.

## 3. Secondary Metabolites of Genus *Acanthella*

Different metabolites were separated and characterized from different species of this genus using various spectroscopic and chromatographic techniques. The isolated metabolites are categorized according to their chemical classes into sesquiterpenes, diterpenes, alkaloids, steroid compounds, and others. Additionally, their reported biosynthetic and synthetic studies are also highlighted whenever applicable.

### 3.1. Sesquiterpenes

**Figure 1:**
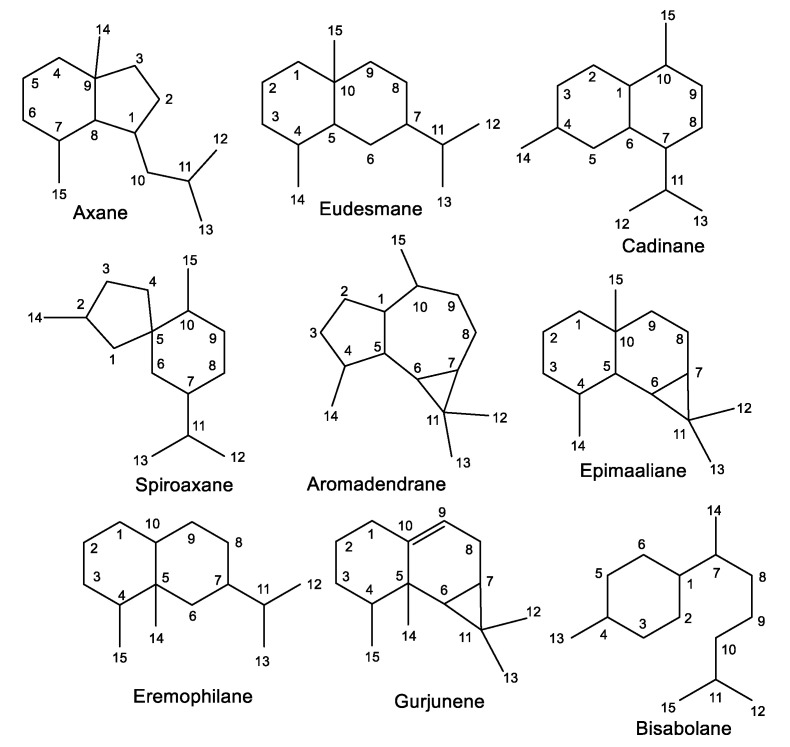
Classes of sesquiterpenes reported from the genus *Acanthella*.

The reported investigations revealed the purification of various classes of sesquiterpenes that are substituted by isonitrile or isothiocyanate functionalities, including mono-, bi-, and tri-cyclic skeletons with 3-, 5-, 6-, and/or 7-membered rings (Figure 1 and Table 2). Frequently, formamide derivatives were reported along with both isothiocyanate and/or isonitrile moieties. Isonitrile-containing metabolites have been reported from some species belonging to *Penicillium* and *Axinella* genera [30]. Several reports stated their characterization from *Acanthella*. It was reported that *A. cavernosa* (Dendy, 1922) can convert cyanide and thiocyanate for isocyanide and isothiocyanate biosynthesis, which could be attributed to the presence of rhodanese or the equivalent enzyme [31]. Therefore, thiocyanate was postulated to be the precursor for the isothiocyanate moiety in terpenes by direct utilization or oxidative desulphurization of cyanide, conversion to isocyanide terpenes, and reinsertion of sulfur [31].

#### 3.1.1. Aromadendrane-Type Sesquiterpenes

In 1987, l-isocyanoaromadendrane (**3**) was reported as a novel isonitrile sesquiterpene from the fish toxic CH_2_Cl_2_ fraction of *A. acuta* using SiO_2_CC (silica gel column chromatography), assigned by spectral and chemical methods [34].

**Figure 2:**
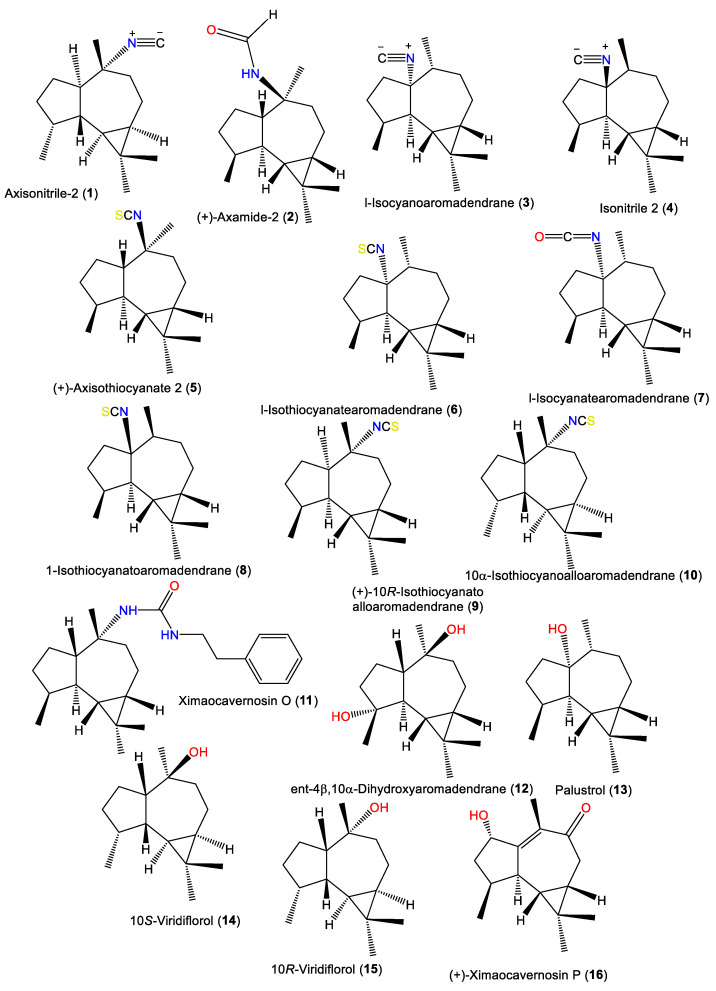
Aromadendrane-type sesquiterpenes (**1**–**16**) reported from the genus *Acanthella*.

Furthermore, **5** and **9** were isolated from Japanese *A. cavernosa* by SiO_2_ CC and RP-HPLC and identified by different spectroscopic methods [40]. Ximaocavernosin O (**11**) was isolated from an *A. cavernosa* Et_2_O fraction using silica gel/MCI/Sephadex LH-20/(RP)-HPLC/chiral-phase HPLC and was characterized by spectroscopy, X-ray diffraction, and QM-NMR analyses as well as Mosher’s method and TDDFT-ECD calculations [33]. Compound **11** is similar to **2** and was formerly reported from nudibranch *Hexabranchus sanguineus* with a C-10 phenyl urea fragment instead of a C-10 formamide in **2** [33]. New aromadendrane-type sesquiterpenoids, **16** and **12**, were purified from the Hainan *A. cavernosa* using SiO_2_/Sephadex LH-20 CC/chiral-phase HPLC by Shen et al.. Their configurations were elucidated based on spectral, TDDFT-ECD, and X-ray analyses and optical rotation measurements. Compound **16** with a 2S/4S/5R/6S/7S configuration ([α]_D_ +169.6]) is identical to 2β-hydroxyaromadendr-1(10)-en-9-one ([α]_D_ ^_^186]) except for the optical rotation (Figure 2) [33].

#### 3.1.2. Spiroaxane-Type Sesquiterpenes

Spiroaxane skeletons containing sesquiterpenes are of rare natural occurrence. Compound **19**, a new sesquiterpene isocyanide with a spiroaxane (spiro [5,6] decane) skeleton was obtained from Chinese *Acanthella* sp., which is a 3-oxo derivative of **17** [41]. Additionally, **23** is a spiroaxane sesquiterpene with a C-6 isocyanate and was purified and characterized by Jumaryatno et al. from *A. cavernosa* specimens collected from Coral gardens/Gneerings reef/Mooloolaba/Australia and from *A. klethra* collected from Pelorus Island, Queensland, in addition to **17** [19,37].

**Figure 3:**
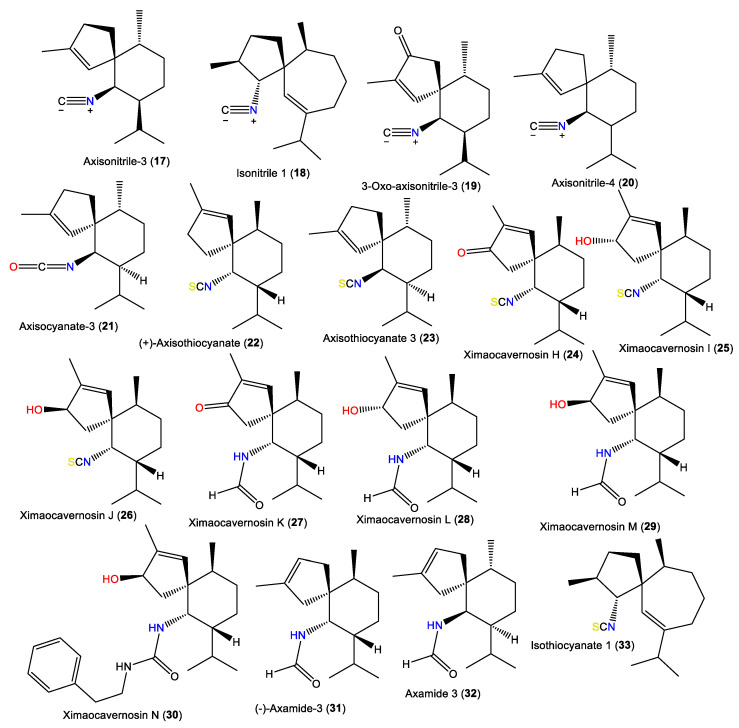
Spiroaxane-type sesquiterpenes (**17**–**33**) reported from the genus *Acanthella*.

Additionally, **23**–**30** were isolated from *A. cavernosa* Et_2_O fractions using silica gel/MCI/Sephadex LH-20/(RP)-HPLC/chiral-phase HPLC and characterized by spectral, X-ray diffraction, and QM-NMR analyses as well as Mosher’s method and TDDFT-ECD calculations [33]. Compounds **23**–**30** are spiroaxane derivatives involving compounds with C-6 isothiocyanate (e.g., **23**–**25**) and formamide or a 1-phenethyl urea fragment (e.g., **26**–**30**) [33] (Figure 3). In 2019, Wu et al. reported the purification of axamide-3 (**32**) from the acetone fraction of *A. cavernosa* collected from Xidao Island (Hainan Province, China) which was characterized by NMR spectral data and optical rotation [48].

#### 3.1.3. Eudesmane-Type Sesquiterpenes

**Figure 4:**
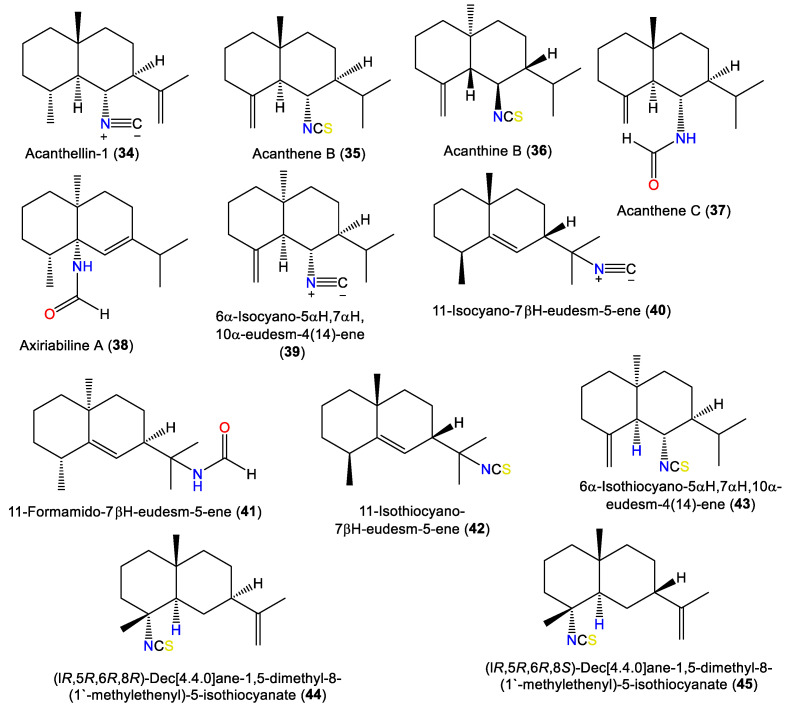
Eudesmane-type sesquiterpenes (**34**–**45**) reported from the genus *Acanthella*.

Acanthellin-1 (**34**) is a bicyclic sesquiterpene with isopropylidene and isonitrile moieties. It was separated as an optically active oil from the ether fraction of the acetone extract of *A. acuta* collected from the Bay of Naples using SiO_2_CC, and was characterized by NMR and chemical methods, as well as optical rotation [30] (Figure 4). A chromatographic investigation of *A. klethra* collected from Pelorus Island, Queensland, yielded sesquiterpenoids with isothiocyanate and isonitrile groups, i.e., **42**, **44**, and **45**, that were assigned by spectral and X-ray analyses. Compounds **42**, **44**, and **45** are of eudesmane-type and are related to **34**. Compounds **45** and **44** are different in stereo-configuration at C-7 [19,44]. Additionally, **39** and **43** are in the bicyclic cis-eudesmane class of sesquiterpenes, possessing isocyanate and isothiocyanate functionalities, respectively, and were purified and specified from *A. acuta* [47], whereas **35** is a stereoisomer of **5** [46].

Axiriabiline A (**38**) was obtained from the acetone fraction of *A. cavernosa* collected from Xidao Island (Hainan Province, China) and characterized by NMR spectral data and optical rotation [48]. Burgoyne et al. (1993) purified two new sesquiterpenoid acanthenes B and C (**35** and **37**) along with **40** and **42**–**44** from the hexane fraction of unidentified *Acantbella* species using SiO_2_ flash CC/HPLC. The compounds were characterized by spectral analyses [46].

#### 3.1.4. Cadinene-Type Sesquiterpenes

**Figure 5:**
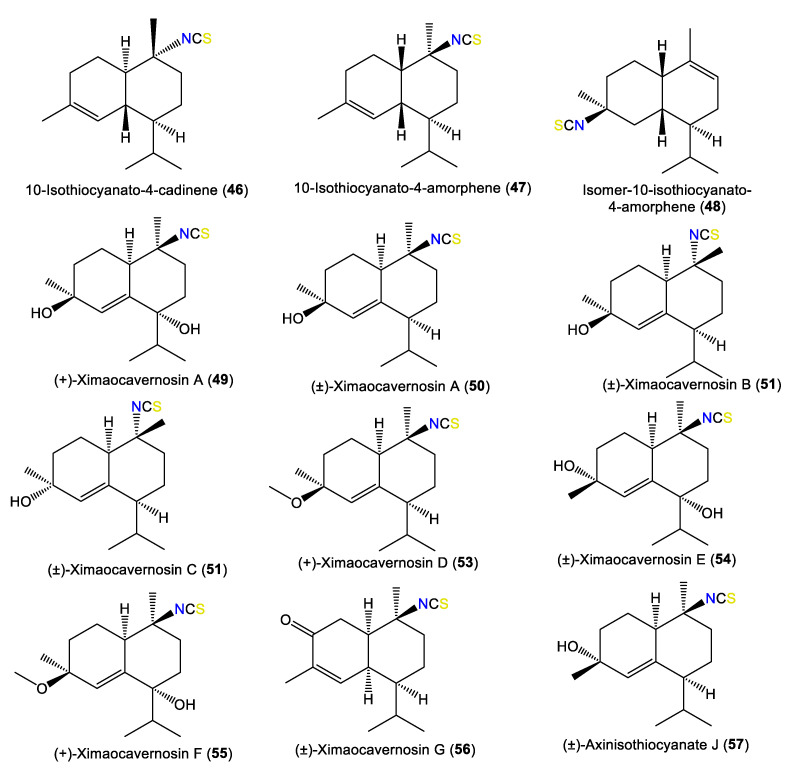
Cadinene-type sesquiterpenes (**46**–**57**) reported from the genus *Acanthella*.

In 2000, Clark et al. isolated a new sesquiterpene, **46**, that has a 1R/6R/7S/10R configuration and C-10 isothiocyanato functionality and [α]_D_ +3 [45]. In addition, Nogata et al. purified a new sesquiterpene, **65**, and the known **67** from *A. cavernosa* EtOH extracts utilizing SiO_2_/Sephadex LH-20/ODS HPLC. These compounds were assigned based on spectral data and chemical transformations [49] (Figure 5). Compound **65** has a C-10 formamido functionality instead of the C-10-OH in **67** [49].

**Figure 6:**
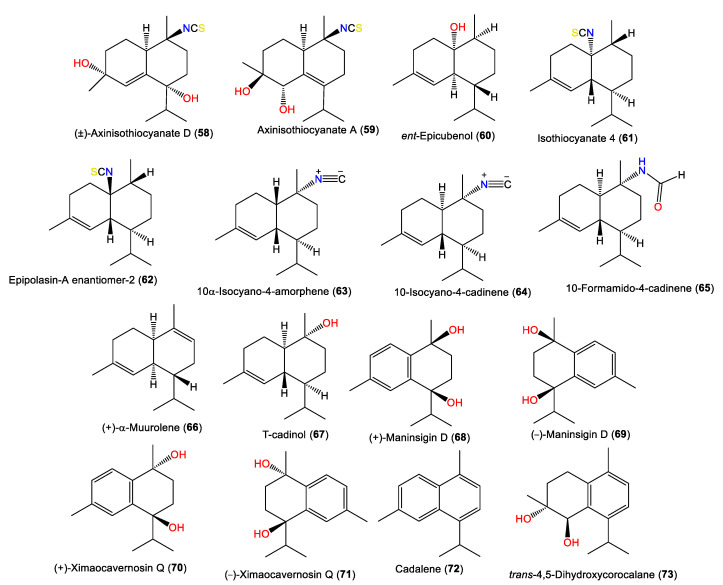
Cadinene-type sesquiterpenes (**58**–**73**) reported from the genus *Acanthella*.

New cadinane-type sesquiterpenoids, ximaocavernosins A–G (**49**–**56**), were isolated from *A. cavernosa* Et_2_O fractions using SiO_2_/MCI/Sephadex LH-20/(RP)-HPLC/chiral-phase HPLC and characterized by spectroscopy, X-ray diffraction, and QM-NMR analyses as well as Mosher’s method and TDDFT-ECD calculations [33]. Compounds **49**–**56** have cadinane frameworks with a Δ^5,6^ double bond and a C-10 isothiocyanate but differ in stereochemistry and oxidation patterns [33]**.** In 2019, Wu et al. reported the purification of 10-formamido-4-cadinene (**65**) from the acetone fraction of *A. cavernosa* collected from Xidao Island (Hainan Province, China), which was characterized by NMR spectral data and optical rotation [48]. New cadinane-type sesquiterpenoids, **49** and **68**–**73**, were purified from Hainan *A. cavernosa* using SiO_2_/Sephadex LH-20 CC/chiral-phase HPLC (Figure 6). Their configurations were elucidated based on spectral, TDDFT-ECD, and X-ray analyses and optical rotation measurements. Maninsigin D and ximaocavernosin Q were obtained as racemic forms, which were separated into their enantiomers [(+)-**68**/(−)-**69** and (+)-**70**/(−)-**71**] using chiral-phase HPLC [43].

#### 3.1.5. Other Sesquiterpenes

**Figure 7:**
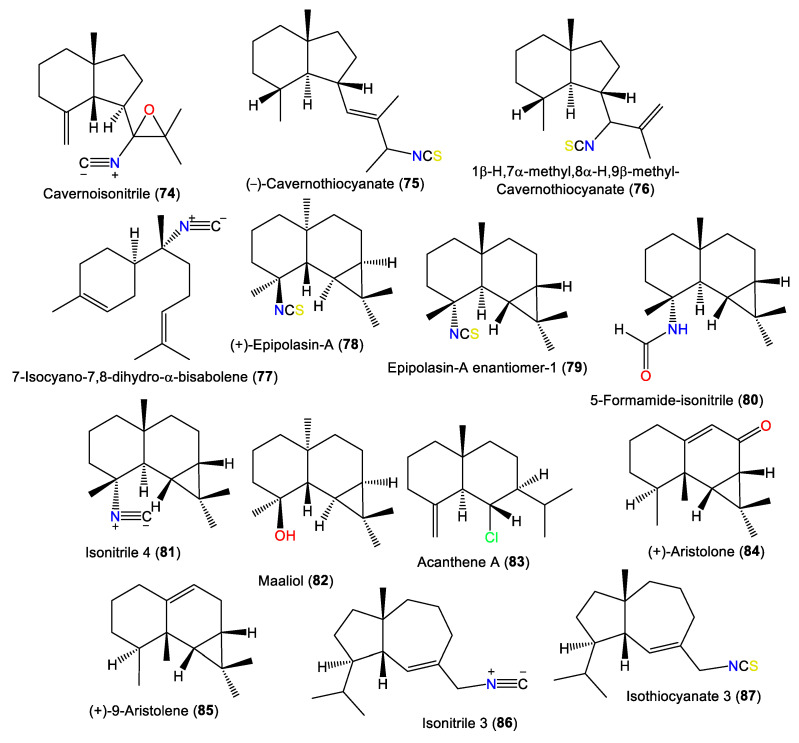
Other sesquiterpenes (**74**–**87**) reported from the genus *Acanthella*.

New axane sesquiterpenoids, **74** and **75**, in addition to **77**, were separated from the antifungal hexane fraction of *A. cavernosa* collected from the Hachijo-Jima Islands using flash CC/sephadex LH-20/HPLC. They were elucidated based on spectral data [23]. Compound **74** is a rare oxygenated tricyclic sesquiterpene cyanide belonging to axane-type sesquiterpenes [23]. Furthermore, **66**, **75, 76**, and **85** were isolated by SiO_2_ CC and RP-HPLC and identified by alpha-D, spectral data, and chemical methods from Japanese *A. cavernosa* [40]. Additionally, the new epimaaliane sesquiterpene **79**, along with **78**, were specified from the antimicrobial acetone extracts of *A. pulcherrima* using spectral and optical rotation measurements. Compound **79** is an enantiomer of **78** with an opposite [α]_D_ value and differs at the ring junction [35]. Burgoyne et al. purified epimaaliane-type sesquiterpenes **80** and **81** from the hexane fraction of an unidentified *Acantbella* species using SiO_2_ flash CC and HPLC. The compounds were characterized by spectral analyses [46] (Figure 7).

**Scheme 1:**
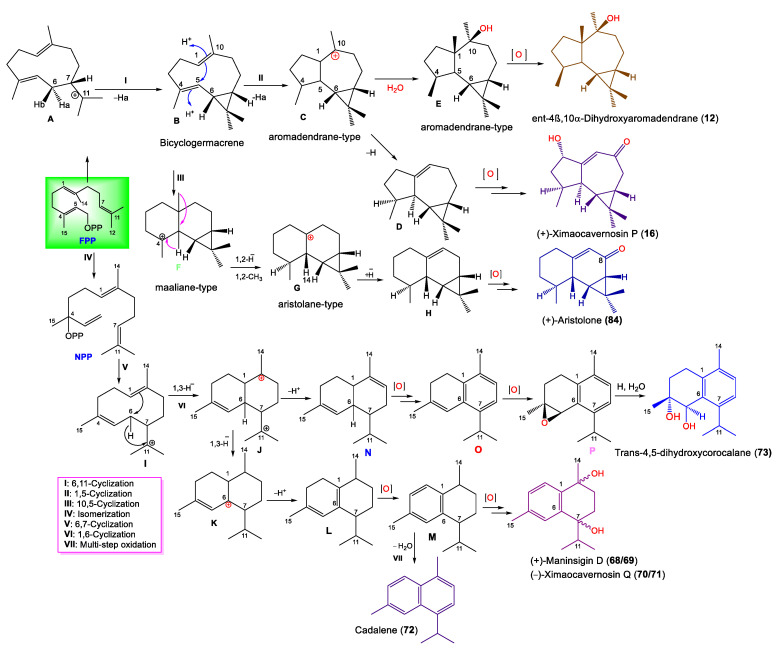
Biosynthesis pathway of **12, 16**, **49**, **68–73**, and **84** [43].

Notably, Shen et al. proposed that **12, 16**, **49**, **68–73**, and **84** originate from E,E-farnesyl diphosphate (E,E-FPP), as illustrated in Scheme 1 [43].

### 3.2. Diterpenoids

**Figure 8:**
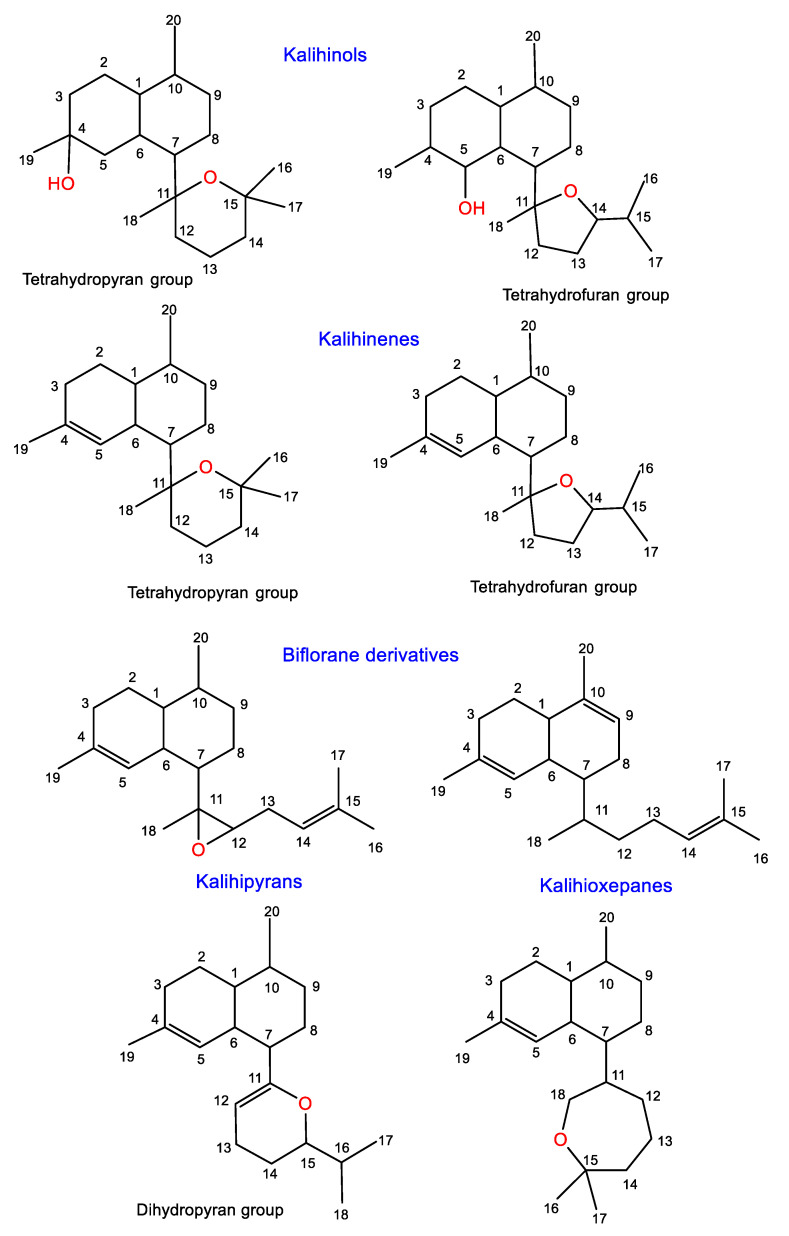
Classes of diterpenes reported from genus *Acanthella*.

Diterpenoids are among the common metabolites reported from various *Acanthella* species. These compounds are characterized by the existence of nitrogenous functionalities such as isothiocyanato, isocyano, and/or formamido groups. These diterpenes are classified into two major classes, kalihinanes and biflorane derivatives, according to the 8C side chain (Figure 8 and Table 3). Kalihinanes have a decalin frame structure with C-7-attached dihydropyran, tetrahydropyran, or tetrahydofuran moiety. Additionally, these rings may carry various substituents such as OH, Cl, isothiocyanato, isocyano, and formamido groups or chlorine. They include kalihinenes, kalihinols, and kalihipyranes. Kalihinols are spilt into two main categories, tetrahydrofuran (**I**) and tetrahydropyran (**II**) groups, according to the C-7 substitution. Commonly, they have *trans*-decalin framework with a C-4 or C-5 tertiary alcohol and an isocyanate moiety at C-10 and/or C-5. The first group has a tetrahydrofuran moiety featuring NCS, NC, or Cl at C-15, or the gem-dimethyl is substituted by an isopropenyl moiety, whereas the tetrahydropyran group possesses Cl atom at C-14. Kalihinenes have a Δ^4^-trisubstituted double bond and possess similar structural features to kalihinols, while biflorane diterpenoids are a class of kalihinane diterpenes featuring a linear eight-carbon open chain substituent at C-7. Biosynthetically, these metabolites are proposed to result from the cyclization of the biflorane skeleton (trans or cis form) and geranylgeranyl pyrophosphate [51]. Their stereochemistry has been determined using spectroscopic, X-ray and/or CD analyses, as well as chemical and computational methods.

#### 3.2.1. Kalihinols

**Figure 9:**
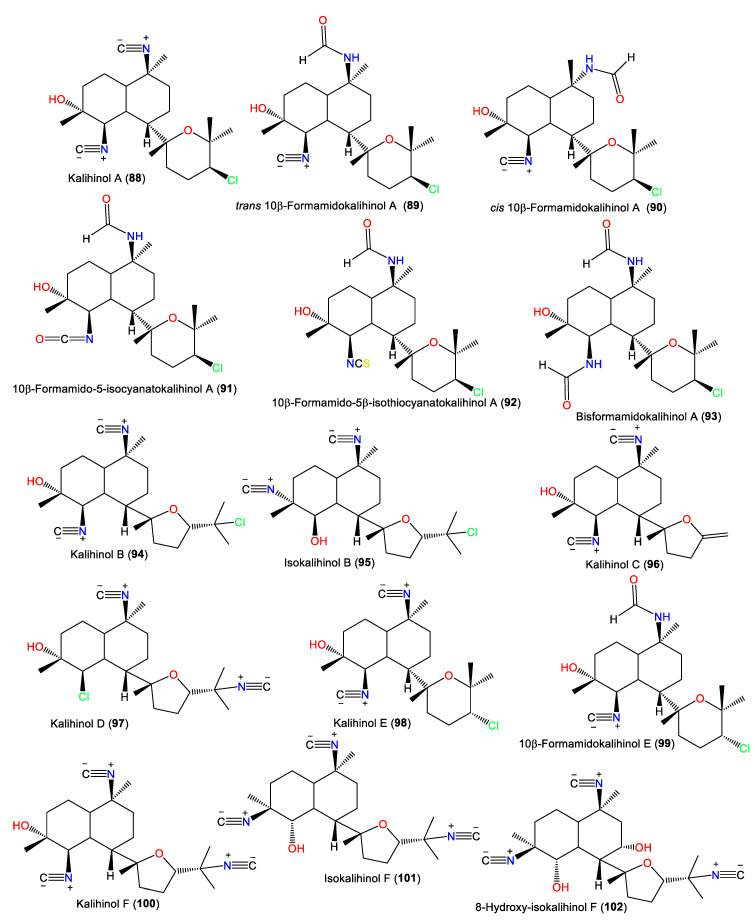
Kalihinol diterpenes (**88**–**102**) reported from the genus *Acanthella*.

Kalihinol A (**88**) was the first reported member of this diterpenoid class in 1984 [52] (Table 3). It is a tricyclic diterpenoid belonging to group **II**, containing isocyano, hydroxyl, and chlorine moieties. It was separated from the CCl_4_ extract of *Acanthella* sp. and characterized by NMR and X-ray analyses [52] and its configuration was assigned as 1S/4R/5R/6S/7S/10S/11R/14S using the CD exciton chirality method [65] (Figure 9).

**Figure 10:**
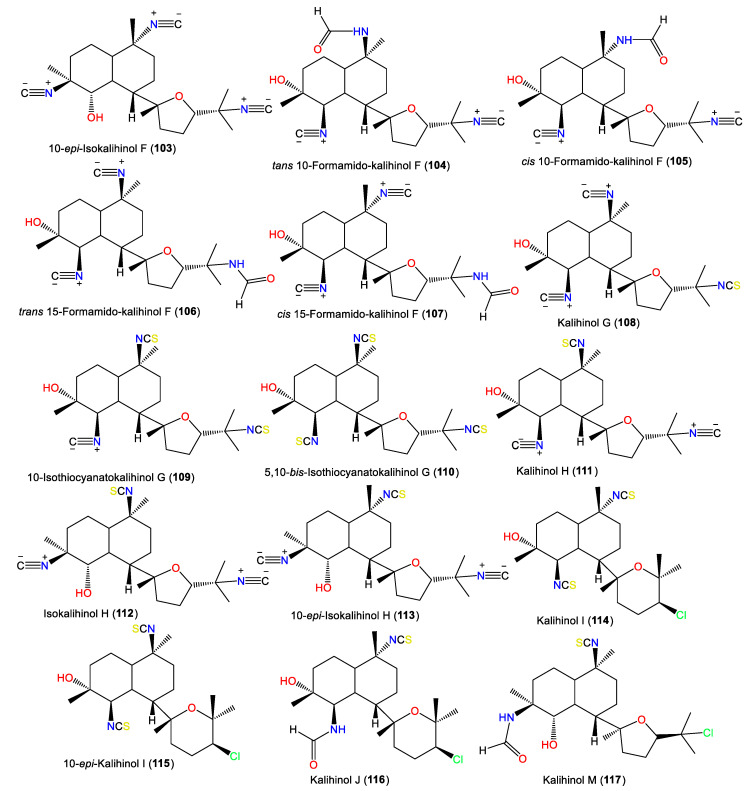
Kalihinol diterpenes (**103**–**117**) reported from the genus *Acanthella*.

In 1987, Chang et al. purified and characterized isocyano-diterpenoids kalihinols A–H (**88**, **94**, **96**–**98**, **100**, **108**, and **111**) and X–Z (**125**, **127**, and **130**) from *Acanthella* sp. obtained from Guam and Fiji using spectral and X-ray analyses. They differ in the C-7 attached moiety; the tetrahydropyran with C-14 chlorine (e.g., **25**, **88**, **98**, **130,** and **127**); or the tetrahydro-furanyl moiety, with 15-NC (e.g., **97** and **100**), 15-NCS (e.g., **108**), 15-C1 (e.g., **94**), or isopropenyl replacing gem-dimethyl (e.g., **96**) [22] (Figure 10).

**Figure 11:**
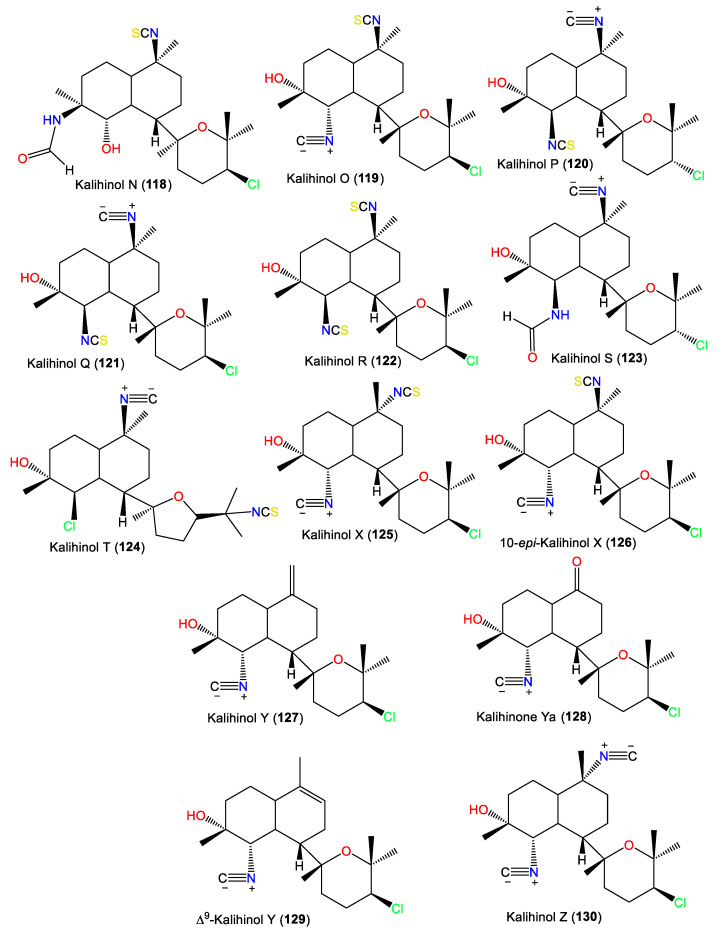
Kalihinol diterpenes (**118**–**130**) reported from the genus *Acanthella*.

Additionally, **101** was isolated as colorless needles from *A. carvenosa* by flash chromatography and HPLC. It resembles **100** with a difference in the substitution at C_4_ and C_5_ [53]. Additionally, new members of the kalihinols family, **114** and **116**, along with **125** and **127**, were purified from *A.cavernosa* collected from Thailand by SiO_2_ CC and HPLC and identified by extensive NMR data. Compound **114** is similar to **125** with a C-5-N-formyl instead of the isothiocyanoate moiety in **125** [18]. Compounds **117** and **118** are two novel C-4 formamido analogs of isokalihinols reported from the South China Sea specimen of *A. cavernosa*. They have a *trans*-decalin ring at C-7 of the tetrahydrofuran and tetrahydropyran rings, respectively. Compound **117** possesses a C-15 chlorine atom and a C-10 isothiocyanato group, whereas **118** is an example of tetrahydropyran-type isokalihinol. They have 1S/4S/5S/6S/7S/10S/11R/14S and 1S/4S/5S/6S/7S/10S/11R/14R configurations, respectively [16]. From Okinawan *Acanthella* sp., new members of kalihinane-type diterpenes, **110**, **115**, and **129**, were purified from the EtOAc fraction using SiO_2_ CC and HPLC. Compound **129** is of tetrahydropyran type and is closely similar to **127**, with a trisubstituted olefinic bond in **129** instead of the exo-methylene group in **127**, while **110** and **115** have three and two isothiocyano groups, respectively [57] (Figure 11).

**Figure 12:**
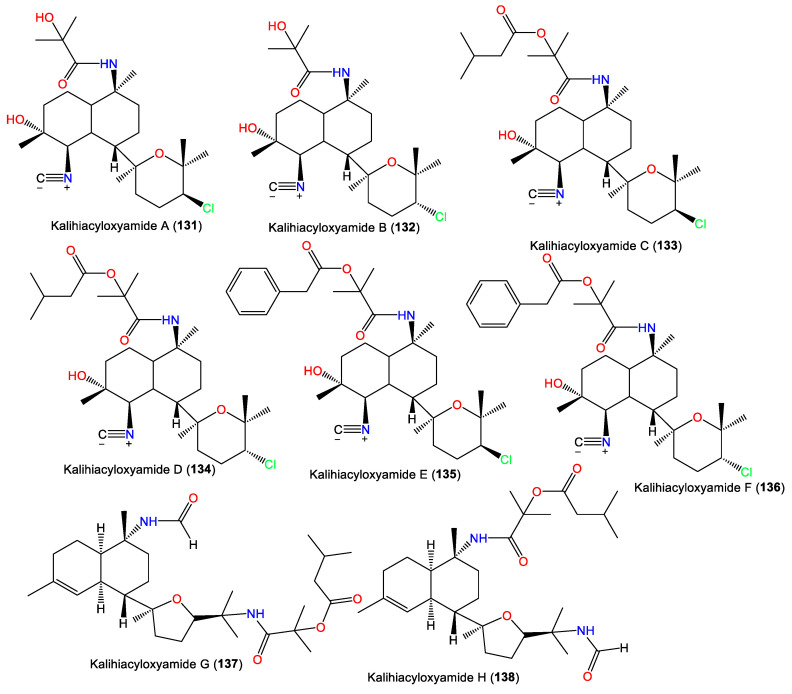
Kalihinol diterpenes (**131**–**138**) reported from the genus *Acanthella*.

In 1994, Trimurtulu et al. reported new diterpene isonitriles, **103** and **113**, from *A. carvenosa* collected from The Seychelles. Compound **103** differs from **101** in the *trans*-decalin ring system configuration, whereas **113** has an isothiocyanate group instead of one of the C-10 isonitrile groups of **103** [59]. Besides, Xu et al. reported new diterpenoids, kalihinols O–T (**119**–**124**), together with **88**, **98**, **109**, **115**, and **126**, from *A. cavernosa* in the South China Sea using SiO_2_ and Sephadex LH-20 CC [16]. Their structures and stereo-structures were determined by NMR/CD/X-ray analyses [16]. Compound **119** is structurally similar to **98**, with a C-10 isothiocyanate instead of a C-10 isonitrile in **98,** whereas **120** is an isocyanato analog of **88** and **123** is a C-5 formamide analog of **126**. Furthermore, **121** and **122** are the C-14 epimers of 120 and 115, respectively, and 124 is the C-15-isothiocyanato analog of **97** [16]. Clark et al. in 2000, purified a new kalihinol-type diterpenoid, 8-OH-isokalihinol F (**102**), from *A. cavernosa* obtained from Heron Island, Great Barrier Reef, Australia, which was structurally similar to **101** with an additional C-8 OH [45]. In addition, new formamide analogs, **104** and **106**, were purified by Bugni et al. in 2004 from two Philippine *A. cavernosa* specimens. They featured formamide moieties at C-10 and C-15, respectively, instead of the isonitrile in **100** [21]. Furthermore, a new kalihinol diterpene, **126**, was isolated from Hainan *Acanthella* sp. by SiO_2_ and Sephadex LH-20 CC and was assigned as a C-10 epimer of **127** [42]. Additionally, new α-acyloxy-amide-substituted diterpenoids, kalihiacyloxyamides A–H (**131**–**138**), were separated from South China Sea *A. cavernosa* EtOAc fractions using SiO_2_/Rp-18 CC/RP-HPLC that were elucidated based on spectral, X-ray, and CD analyses (Figure 12). These metabolites featured isobutyl amide (e.g., **131** and **132**), iso-amyl ester (e.g., **133**, **134**, **137**, and **138**), and phenethyl ester (e.g., **135** and **136**) groups [62].

#### 3.2.2. Kalihinenes

**Figure 13:**
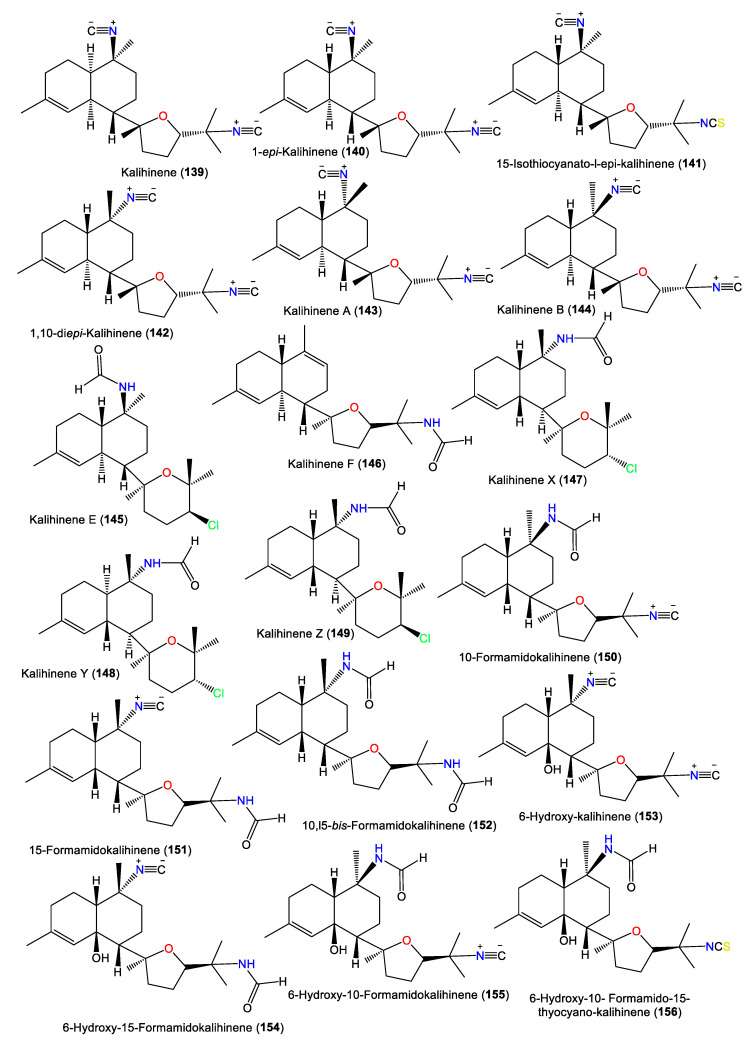
Kalihinene diterpenes (**139**–**156**) reported from the genus *Acanthella*.

The first member of this group is kalihinene (**139**), which was purified from an *A. klethra* EtOH extract using SiO_2_ CC/Develosil ODS-5 CC/HPLC and assigned by NMR and X-ray analyses [23]. Furthermore, compounds **143** and **144** were reported as novel monounsaturated kalihinane class diterpenes derived from *A. Cavernosa* toxic CH_2_Cl_2_ extracts against *Artemia salina* and *Lebistes reticulatus* using VLC/Flash/Rp-18 CC. These two compounds are diastereoisomers of **139** that feature a trans-decalin skeleton instead of the trans-decalin skeleton of kalihinene. On the other hand, **143** and **144** are epimers at C-10 [54]. Additionally, **145** and **146** are tertrahydropyran/trans-decalin and tetrahydrofuran/cis-decalin analogs bearing formamido groups at C-10 and C-15, respectively (Figure 13). Kalihinene E (**145**) is a C-14 epimer of **148** with 1*S*/6*S*/7*S*/10*S*/11*R*/14*R* configuration [63]. In 1994, Rodríguez et al. purified new diterpenoids belonging to kalihinene and 6-OH kalihinene groups (**148** and **150**–**156**), along with **139** from *A. cavernosa* collected from a Fijian location. These compounds were characterized based on spectral and X-ray analyses as well as biogenetic evidence [51]. Additionally, **140**–**142** are new metabolites reported from *A. carvenosa* collected from The Seychelles [59]. Compound **140** is a C-1 isomer of **139**, **141** has C-15 isothiocyanate instead of C-15 isonitrile in **140**, and **142** is an isomer of **140** and **139** [59].

#### 3.2.3. Kalihipyrans and Kalihioxepanes

**Figure 14:**
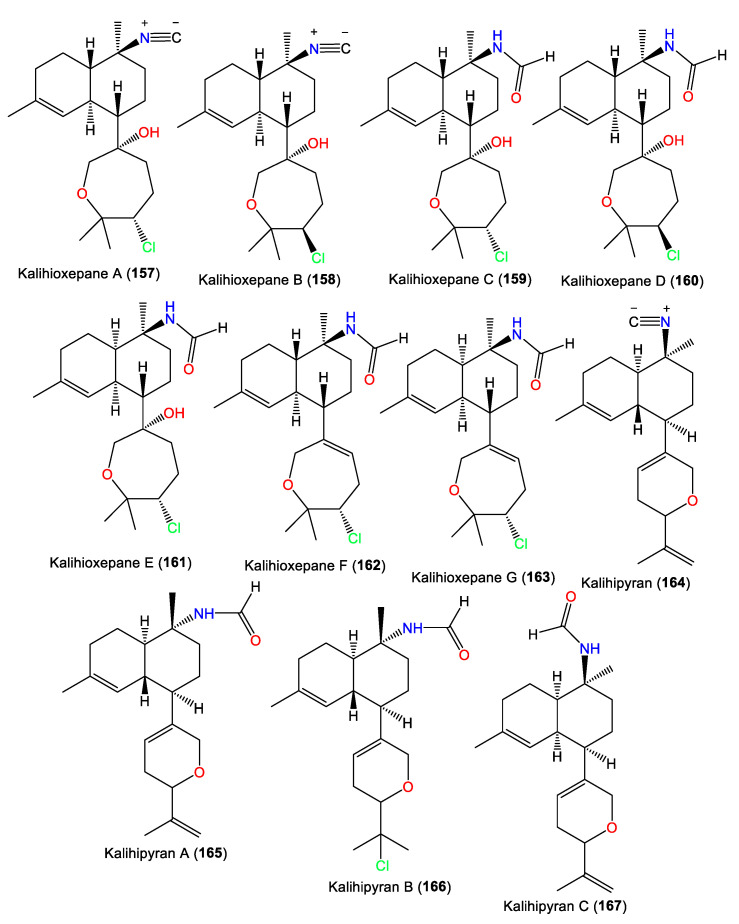
Kalihioxepanes (**157**–**163**) and kalihipyrans (**164**–**167**) diterpenes reported from the genus *Acanthella*.

Kalihipyran (**164**) is a tricyclic kalihinene-type diterpene with a C-7 isopropenyl-containing dihydropyran moiety and a C-10 isonitrile [59]. Compound **167** is an isomer of **165** with cis-decalin [63], while **166** has a C-15 chlorine atom [55,56]. In 2022, Wang et al. purified new kalihinane diterpenoids, kalihioxepanes A–G (**157–163**), from South China Sea *A. cavernosa* by the means of SiO_2_/Sephadex LH-20/HPLC. The structures were elucidated by spectral and X-ray analyses, in addition to quantum chemical calculation methods [64] (Figure 14).

**Scheme 2:**
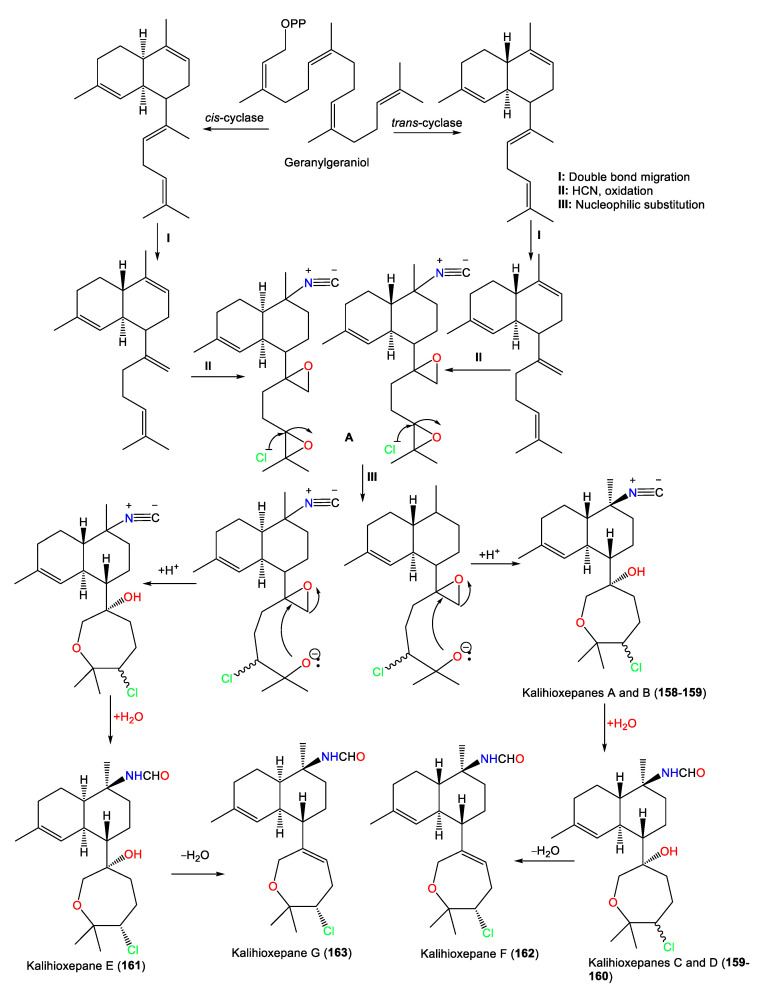
Biosynthesis of kalihioxepanes A–G (**157**–**163**) [64].

These metabolites possess a rare C-7-attached oxepane ring with a C-14 Cl atom. Compounds **157**–**160** have a *trans* decalin skeleton; however, **161**–**163** have a cis-decalin skeleton with C-10 isonitrile (e.g., **157** and **158**) and formamide (e.g., **159**–**163**) groups [64]. Wang et al. proposed that **157**–**163** are biosynthesized using geranylgeraniol as a precursor (Scheme 2). The latter undergoes a series of reactions, including cyclization, double-bond migration, nucleophilic addition, and oxidation reactions, to give epoxide biflorane (**A**, the key intermediate). Nucleophilic substitution of biflorane forms **157** and **158**. After that, hydration generates **159**–**161** which are then dehydrated to give **162** and **163**, respectively [64].

#### 3.2.4. Biflorane Diterpenes

**Figure 15:**
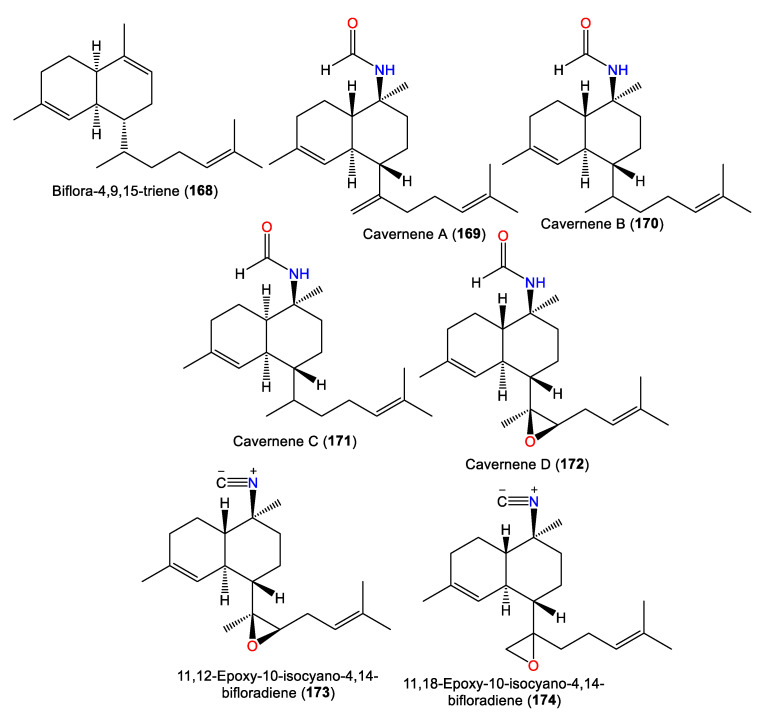
Biflorane (**168**–**174**) diterpenes reported from the genus *Acanthella*.

From the Japanese *A. cavernosa*, biflora-4,9,15-triene (**168**) was separated, which is a rare biflorane diterpene related to **66**, by replacing the methyl hydrogen of the isopropyl group of **66** with a prenyl group [40]. In 2012, Xu et al. reported **169–172** from CH_2_Cl_2_ extracts of South China Sea *A. cavernosa*, bearing a C-10 formamide group that varied in the decalin moiety (*cis* or *trans*) configuration and nature of C-7-linked side chain [63] (Figure 15). Their structures were assigned by spectral and X-ray analyses. Compounds **169**, **170**, and **172** are *trans*-decalin derivatives, with a C-7 isoprenoid unit, a mono-olefinic isoprenoid sidechain, and a trisubstituted epoxide in the side chain, respectively. In contrast, **171** had a *cis*-decalin moiety [63].

**Scheme 3:**
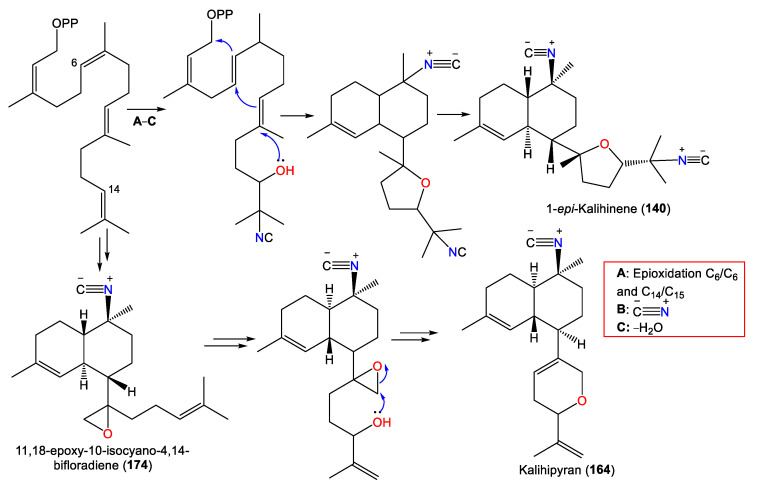
Biosynthesis of **140**, **164**, and **174** [45].

Investigation of *A. cavernosa* DCM/MeOH extracts led to the separation of two oxirane analogs with a trans-decalin framework, **173** and **174**, featuring a trisubstituted epoxide and a terminal epoxide group in the side chain, respectively. Compound **174** was suggested to be a precursor of the kalihipyran skeleton [45]. Clark et al. proposed that the biosynthesis of pyranyl and furanyl kalihinols involves epoxidation of the bifloradiene precursor’s terminal double bond by a nucleophilic attack at either epoxide end by a cyanide ion to form a hydroxyisocyanide. The latter initiates cyclisation to afford a bicyclic system (Scheme 3). Compounds **173** and **174** are alternative epoxidation products. Compound **174** was suggested to be a precursor of the kalihipyran skeleton [45].

### 3.3. Alkaloids

Several reports have stated the isolation of different classes of alkaloids from this genus. It is noteworthy that bromopyrrole alkaloids are the dominant type reported from the species of this genus (Table 4). Oroidin **177** is the first member of pyrrole 2-aminoimidazole alkaloids. These alkaloids were reported to have significant bioactivities, as well as chemical defense against predator fish.

**Scheme 4:**
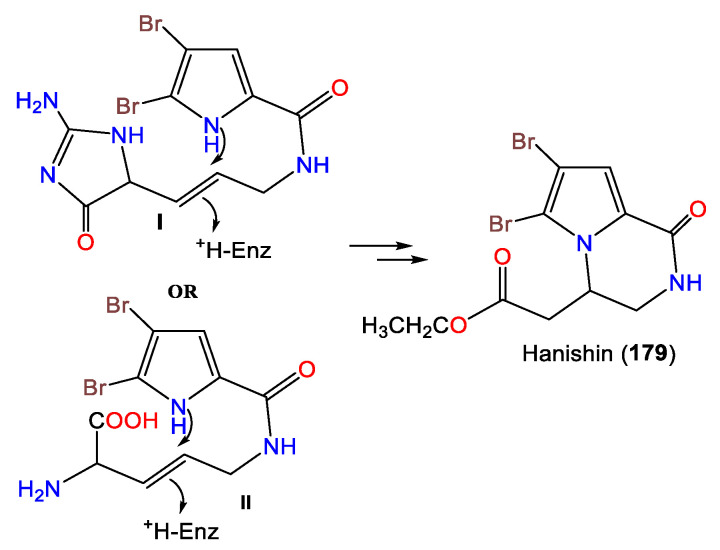
Proposed biosynthesis of **179** [66].

In 2010, Hammami et al. purified a novel bromopyrolimidazole analog, **178**, along with **177** from Tunisian *A. acuta* diethyl ether extracts [25]. Four bromo-pyrrole alkaloids, including novel alkaloid hanishin (**179**) in addition to **175**–**177**, were isolated from *A. carteri* collected from the northern coast of Hanish Island, Yemen, South Red Sea, by Mancini et al. Compounds **177** and **179** are members of the oroidin family of alkaloids that are considered condensation products of prolines. Compound **179** was proposed to be derived from aminoimidazolinone (**I**) or amino acid (**II**) intermediates through 1N-C9 cyclization with subsequent side-chain oxidative breakdown [66] (Scheme 4).

**Figure 16:**
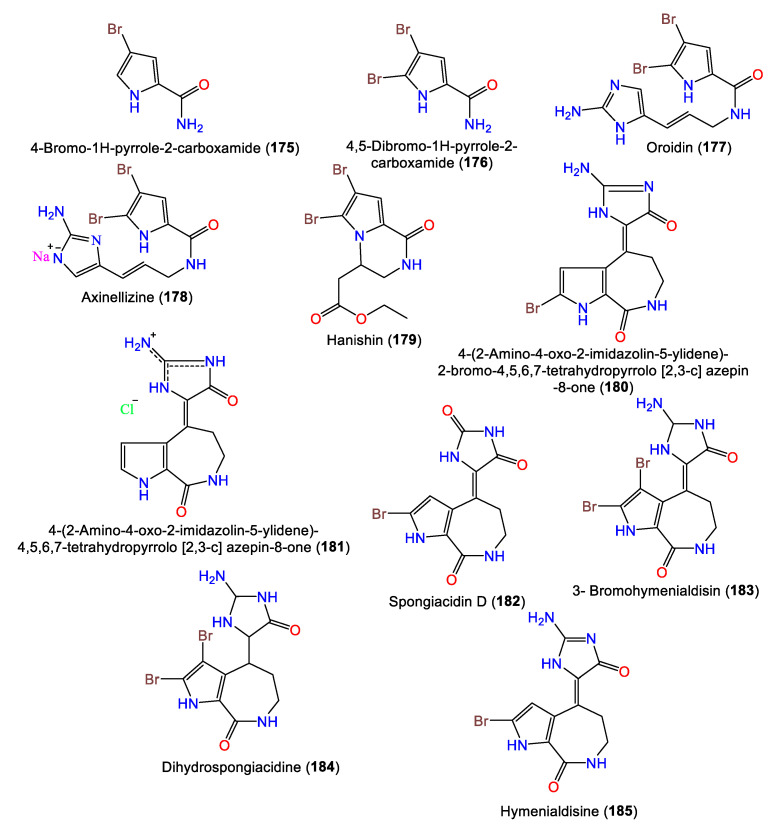
Alkaloids (**175**–**185**) reported from genus *Acanthella*.

Mattia et al. purified **180** as a brominated alkaloid from Red Sea *A. Aurantiaca* BuOH extracts. The compound features an aminooxodihydroimidazole ring linked to a pyrroloazepine group via a double bond (Figure 16) [68]. Compounds **180** and **181** were obtained from *A. aurantiaca* BuOH extracts using Sephadex LH-20 and crystallization and were characterized by spectral and X-ray analyses [67]. In 2014, Macabeo and Guce reported the bromopyrrole-imidazole alkaloids **182**–**184** from CH_2_Cl_2_-MeOH extracts of *A. carteri* from The Philippines [17], while **185** is a pyrrole alkaloid isolated from the n-BuOH fraction of *Acanthella* sp. using Sephadex LH-20/Rp-18 CC [69].

**Scheme 5:**
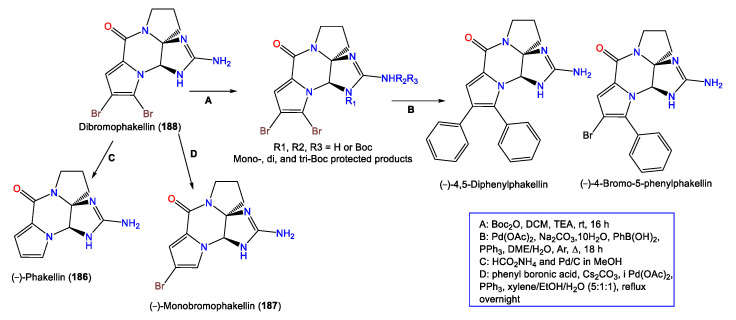
Semisynthesis of (−)-dibromophakellin (**188**) derivatives [70].

A series of synthetic reactions including Suzuki–Miyaura coupling and debromination resulted in natural analogs **186** and **187**, in addition to new synthetic derivatives (−)-4-bromo-5-phenylphakellin and (−)-4,5-diphenylphakellin. It was found that the C-5 Br substitution with phenyl or H led to a loss in activity, revealing that the C-5 Br is important for α2B adrenoceptor agonistic activity (Scheme 5) [70].

Furthermore, **190** was purified from *A. carteri* using Sephadex LH-20/SiO_2_ CC, giving a bright-orange color with a diazotized benzidine. The compound was characterized by NMR and X-ray analyses, as well as chemical methods. Compound **190** is a 6R/10S brominated alkaloid with a fused C-C pyrrole linkage to the cyclic guanidine core belonging to the **189** series [71].

**Scheme 6:**
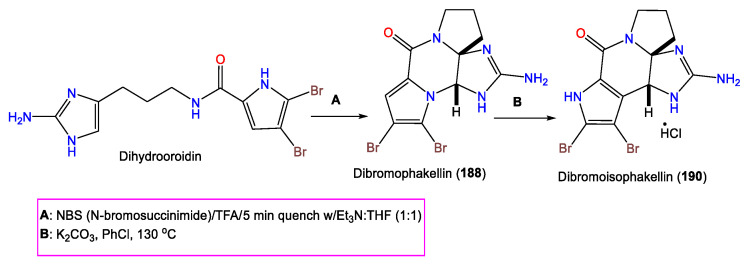
Synthesis of **190** using dihydrooroidin [73].

In 2002, Wiese and his group reported the synthesis of **190** using dihydrooroidin that is converted to **188** (Scheme 6). Then, thermal rearrangement of **188** in the presence of K_2_CO_3_ produces **190** [73].

**Figure 17:**
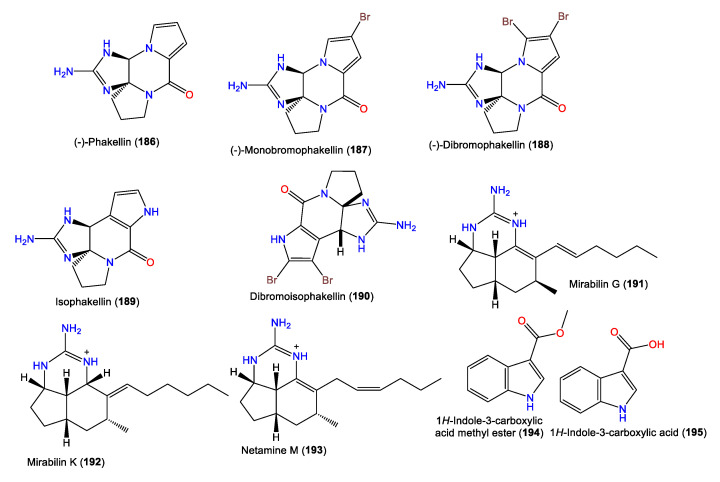
Alkaloids (**186**–**195**) reported from the genus *Acanthella*.

Additionally, Grkovic et al. were able to separate tricyclic-guanidine-containing alkaloids, including a new analog mirabilin K (**192**), along with **191** and **193**, from *A. cavernosa* collected in Southwestern Australia using diol flash chromatography/Rp-18/HPLC. The compounds were characterized by spectroscopic analyses and optical rotation measurements. Compound **192** has a 4S*/7S*/9R*/11S*/12R* configuration, which differs from **191** in the C9-CH_3_ group and with the presence of a N-substituted methine group (Figure 17) [24]. Furthermore, **194** and **195** were obtained by Fan et al. from the acetone extracts of *A. cavernosa* collected from the South China Sea [50].

**Figure 18:**
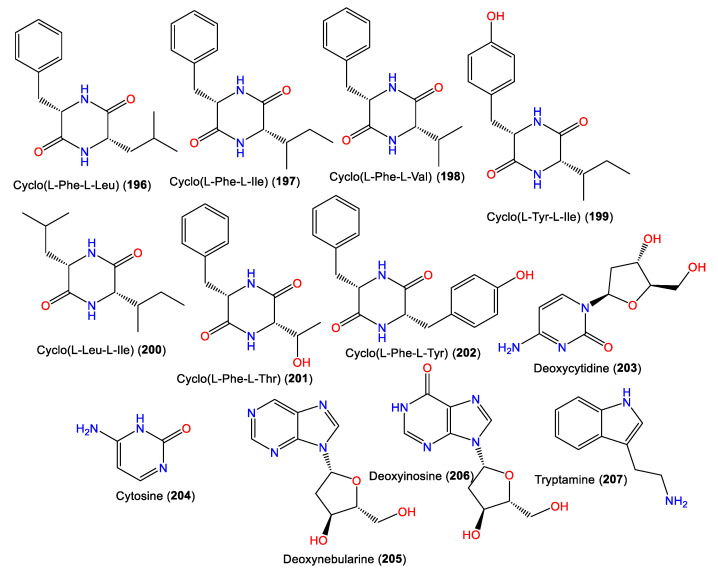
Alkaloids (**196**–**207**) reported from the genus *Acanthella*.

Diketopiperazines, including the rare cyclo(L-Phe-L-Thr) and cyclo(L-Tyr-L-Ile) (**196**–**202**), along with decarboxylated amino acid **207** and deoxyribonucleotides **203**–**206**, were reported and characterized from Fijian *A. cavernosa* (Figure 18). Their L-L absolute configuration was assigned based on an NMR and CD comparison with synthetic L-L analogs, as well as optical rotation measurements [72].

### 3.4. Steroid Compounds

**Figure 19:**
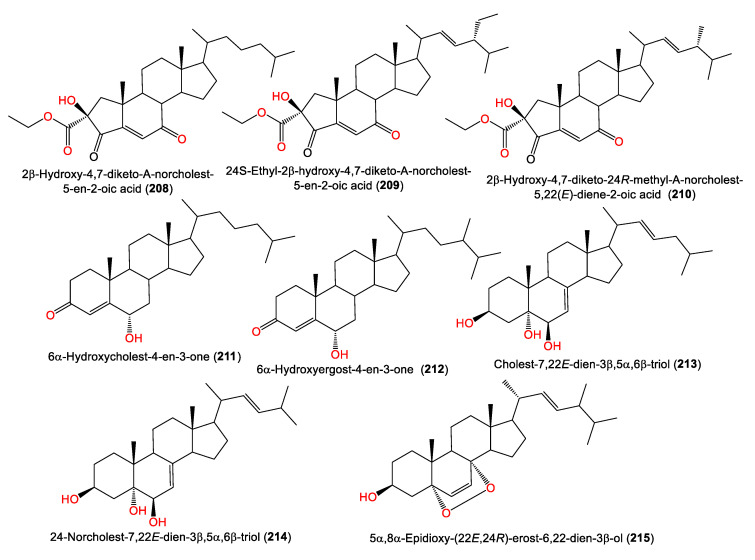
Steroid compounds (**208**–**215**) reported from the genus *Acanthella*.

In 2008, Qui et al. reported the purification of three new nor-steroids, **208**–**210**, along with the known steroids **211**–**214** from the petroleum ether fraction of *A. cavernosa* obtained from Hainan Island, China, using SiO_2_ CC/HPLC. The new steroids are related to A-ring-contracted steroid analogs featuring carbonyl and ketone groups located at C-3 and C-4; they differ in their C-17 side chains [74] (Table 5 and Figure 19). In addition, **215** was obtained from the acetone extract of the same sponge collected from the South China Sea [50].

### 3.5. Other Metabolites

**Figure 20:**
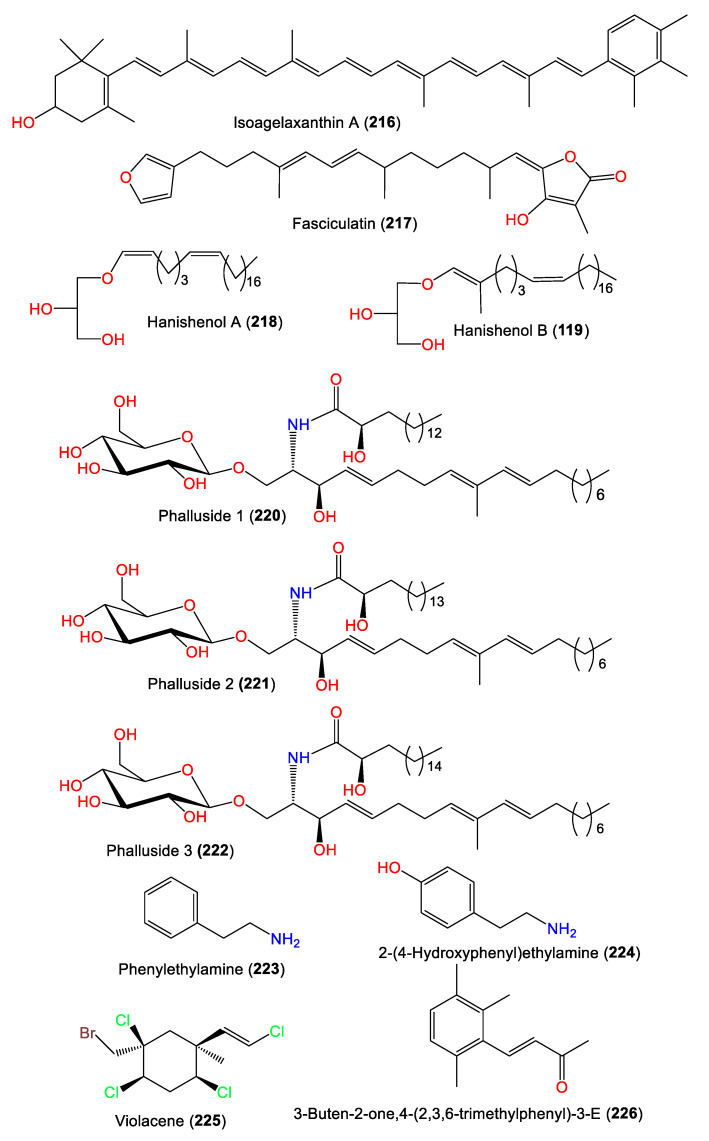
Other metabolites (**216**–**226**) reported from the genus *Acanthella*.

Compound **216** was separated from *A. vulgata* acetone extracts using an MgO column and crystallization from petroleum ether. The compound belongs to carotenoids, as it has a polyene chain with terminal aromatic moieties on both ends [75] (Figure 20). Mancini et al. were able to purify and characterize **219**, a novel methyl-branched glycerol enol ether, and the related linear analog **218** from *A. carteri* obtained from Southern Red Sea Hanish Islands by utilizing flash CC/HPLC and spectral and chemical methods [76]. Compound **219** has an additional methyl group at C-2 of the sidechain compared to **218**, and both have a 2`S configuration [76]. In 2010, Hammami et al. separated the sesterterpene **217** and cerebrosides **220**–**222** from Tunisian *A. acuta* diethyl ether extracts [25], whereas **226** was purified from the Chinese *A. cavernosa* by Fan et al. [50].

## 4. Biological Activities of *Acanthella* Species and Their Metabolites

Various *Acanthella* species and their metabolites have been found to display various bioactivities. The reported investigations are highlighted below, and some results are listed in Table 6.

### 4.1. Antimicrobial and Antifouling Activities

McCaffrey and Endean reported that *A. kleutha’s* toluene/methanol (1:3 v/v) and CH_2_Cl_2_ extracts displayed antimicrobial potential comparable with penicillin G and streptomycin versus *B. subtilis*, *K. pneumoniae*, and *S. aureus* [77]. The *A. carteri* MeOH extract that was collected from Ras Nusrani in the Gulf of Aqaba was significantly effective versus *B. subtilis*, *S. aureus*, *P. vulgaris*, *E. coli*, *C*. *tropicalis*, and *C. albicans* (inhibition zone 9.0–23.3 mm) [78]. On the other hand, the n-BuOH fractions of *A. acuta* showed more promising antimicrobial potential versus *A. niger*, *C. albicans*, and *S. aureus* than CH_3_Cl fractions [79]. The MeOH extract of *Acanthella elongata* caused 100% and 87.5% inhibition of marine fish pathogens *Aeromonas hydrophila*, *Pseudomonas aeruginosa*, *Vibrio alginolyticus*, *V. anguillarum*, *V. fischeri*, *V. fluvialis*, *V. pelagius*, and *V*. *vulnificus* at 30°C and 20°C [20]. Additionally, *A. cavernosa* and *A. ramosa* from the Bay of Bengal exhibited activity versus the virulent fish pathogens *Edwardsiella tarda*, *A. hydrophila*, *P. aeruginosa*, *V. alginolyticus*, and *P. fluorescens* [80]. Rajendran et al. reported that the *A. elongata* CH_2_Cl_2_ fraction had antimicrobial properties versus *V. alginolyticus* (fish pathogen) and *R. solani*, while the EtOH extract prohibited *Vibrio fisherii* and *Micrococcus* sp. [81]. Nogata et al. reported that among the tested 86 Japanese sponge species, the EtOH extract of *A. cavernosa* obtained from Atami, Shizuoka prefecture, Tokyo, displayed 100% inhibition of *B. amphitrite* larval settlements with no toxicity [49]. Additionally, Okino et al. reported that the EtOH extract of *A. cavernosa* obtained from Yakushima Island had antifouling potential as it prohibited metamorphosis and larval settlement of the *Balanus amphitrite* barnacle [56].

**Table 6:** Biological activity of reported metabolites from the genus *Acanthella*.

Compound Name	Biological Activity	Assay, Organism or Cell Line	Biological Results		Ref.
			Compound	Positive Control	
(+)-10(*R*)-Isothiocyanatoalloaromadendrane (**9**)	Cytotoxicity	MTT/A549	1.98 µg/mL (IC_50_)	-	[42]
Axisonitrile-3 (**17**)	Antimalarial	*Plasmodium falciparum*-D6/Microculture radioisotope	142.0 ng/mL (IC_50_)	Chloroquine 1.95 ng/mL (IC_50_)	[19]
		*Plasmodium falciparum*-W2/Microculture radioisotope	16.5 ng/mL (IC_50_)	Chloroquine 22.8 ng/mL (IC_50_)	[19]
	Cytotoxicity	MTT/A549	2.44 µg/mL (IC_50_)	-	[42]
11-Isothiocyano-7βH-eudesm-5-ene (**42**)	Antimalarial	*Plasmodium falciparum*-D6/Microculture radioisotope	2240.0 ng/mL (IC_50_)	Chloroquine 1.95 ng/mL (IC_50_)	[19]
		*Plasmodium falciparum*-W2/Microculture radioisotope	610.0 ng/mL (IC_50_)	Chloroquine 22.8 ng/mL (IC_50_)	[19]
(l*R*,5*R*,6*R*,8*S*)-Dec[4.4.0]ane-1,5-dimethyl-8-(1′-methylethenyl)-5-isothiocyanate (**45**)	Antimalarial	*Plasmodium falciparum*-D6/Microculture radioisotope	4000.0 ng/mL (IC_50_)	Chloroquine 1.95 ng/mL (IC_50_)	[19]
		*Plasmodium falciparum*-W2/Microculture radioisotope	550.0 ng/mL (IC_50_)	Chloroquine 22.8 ng/mL (IC_50_)	[19]
Kalihinol A (**88**)	Antibacterial	Microbroth dilution/*B. subtilis* PY79	50.0 μg/mL (MIC)	-Trimethoprim 1.0 μg/mL (MIC) -Ciprofloxacin 1.0 μg/mL (MIC) -Rifampin 1.0 μg/mL (MIC) -Sulfamethoxazole 4.0 μg/mL (MIC) -Chloramphenicol 16.0 μg/mL (MIC) -Polymyxin B 16.0 μg/mL (MIC)	[21]
		Disc-diffusion/*Ruegeria CtaxMed-2*	0.67 mm (IZD) at 10 μg/Disc	Streptomycin 0.83 mm (IZD) at 10 μg/Disc	[58]
		Disc-diffusion/*Vibrio sp. (NAP-4)*	1.0 mm (IZD) at 10 μg/Disc	Streptomycin 1.83 mm (IZD) at 10 μg/Disc	[58]
		Disc-diffusion/*Vibrio furnissii*	6.33 mm (IZD) at 10 μg/Disc	Streptomycin 2.50 mm (IZD) at 10 μg/Disc	[58]
	Antifouling	Settlement inhibition/*Balanus amphitrite*	0.087 µg/mL (IC_50_)	CuSO_4_ 0.15 µg/mL (IC_50_)	[55,56]
		Settlement inhibition/*Balanus amphitrite*	0.92 µM (EC_50_)	Seawater + DMSO	[16]
	Cytotoxicity	MTT/HCT-116	17.40 µM (IC_50_)	-	[16]
*trans* and *cis* 10β-Formamidokalihinol A (**89/90**)	Antibacterial	Disc-diffusion/*Vibrio sp.* (NAP-4)	1.0 mm (IZD) at 10 μg/Disc	Streptomycin 1.83 mm (IZD) at 10 μg/Disc	[58]
		Disc-diffusion/*Vibrio furnissii*	5.67 mm (IZD) at 10 μg/Disc	Streptomycin 2.50 mm (IZD) at 10 μg/Disc	[58]
	Antifouling	Settlement inhibition/*Balanus amphitrite*	1.37 µM (EC_50_)	Seawater + DMSO	[16]
	Cytotoxicity	MTT/CT-26	28.82 µM (IC_50_)	-	[16]
10β-Formamido-5β-isothiocyanatokalihinol A (**92**)	Antifouling	Settlement inhibition/*Balanus amphitrite*	0.41 µM (EC_50_)	Seawater + DMSO	[16]
Isokalihinol B (**95**)	Cytotoxicity	MTT/P388	0.8 μg/mL	-	[23]
Kalihinol D (**97**)	Cytotoxicity	MTT/A549	3.17 μg/mL (IC_50_)	-	[42]
Kalihinol E (**98**)	Antifouling	Settlement inhibition/*Balanus amphitrite*	1.85 µM (EC_50_)	Seawater + DMSO	[16]
	Cytotoxicity	MTT/HCT-116	18.31 µM (IC_50_)	-	[16]
Kalihinol F (**100**)	Antibacterial	Microbroth dilution/*B. subtilis* PY79	12.5 μg/mL (MIC)	-Trimethoprim 1.0 μg/mL (MIC) -Ciprofloxacin 1.0 μg/mL (MIC) -Rifampin 1.0 μg/mL (MIC) -Sulfamethoxazole 4.0 μg/mL (MIC) -Chloramphenicol 16.0 μg/mL (MIC) -Polymyxin B 16.0 μg/mL (MIC)	[21]
*Trans* 10-Formamido-kalihinol F (**104/105**)	Antibacterial	Microbroth dilution/*B. subtilis* PY79	50.0 μg/mL (MIC)	-Trimethoprim 1.0 μg/mL (MIC) -Ciprofloxacin 1.0 μg/mL (MIC) -Rifampin 1.0 μg/mL (MIC) -Sulfamethoxazole 4.0 μg/mL (MIC) -Chloramphenicol 16.0 μg/mL (MIC) -Polymyxin B 16.0 μg/mL (MIC)	[21]
15-Formamido-kalihinol F (**106/107**)	Antibacterial	Microbroth dilution/*B. subtilis* PY79	50.0 μg/mL (MIC)	-Trimethoprim 1.0 μg/mL (MIC) -Ciprofloxacin 1.0 μg/mL (MIC) -Rifampin 1.0 μg/mL (MIC) -Sulfamethoxazole 4.0 μg/mL (MIC) -Chloramphenicol 16.0 μg/mL (MIC) -Polymyxin B 16.0 μg/mL (MIC)	[21]
Kalihinol G (**108**)	Antibacterial	Microbroth dilution/*B. subtilis* PY79	3.12 μg/mL (MIC)	-Trimethoprim 1.0 μg/mL (MIC) -Ciprofloxacin 1.0 μg/mL (MIC) -Rifampin 1.0 μg/mL (MIC) -Sulfamethoxazole 4.0 μg/mL (MIC) -Chloramphenicol 16.0 μg/mL (MIC) -Polymyxin B 16.0 μg/mL (MIC)	[21]
10-*epi*-Kalihinol I (**115**)	Antifouling	Settlement inhibition/*Balanus amphitrite*	0.27 µM (EC_50_)	Seawater + DMSO	[16]
	Cytotoxicity	MTT/HCT-116	28.67 µM (IC_50_)	-	[16]
Kalihinol J (**116**)	Antibacterial	Microbroth dilution/*B. subtilis* PY79	50.0 μg/mL (MIC)	-Trimethoprim 1.0 μg/mL (MIC) -Ciprofloxacin 1.0 μg/mL (MIC) -Rifampin 1.0 μg/mL (MIC) -Sulfamethoxazole 4.0 μg/mL (MIC) -Chloramphenicol 16.0 μg/mL (MIC) -Polymyxin B 16.0 μg/mL (MIC)	[21]
Kalihinol O (**119**)	Antifouling	Settlement inhibition/*Balanus amphitrite*	1.43 µM (EC_50_)	Seawater + DMSO	[16]
	Cytotoxicity	MTT/HCT-116	5.97 µM (IC_50_)	-	[16]
Kalihinol P (**120**)	Antifouling	Settlement inhibition/*Balanus amphitrite*	0.72 µM (EC_50_)	Seawater + DMSO	[16]
	Cytotoxicity	MTT/HCT-116	10.68 µM (IC_50_)	-	[16]
		MTT/H1299	26.21 µM (IC_50_)	-	[16]
Kalihinol Q (**121**)	Antifouling	Settlement inhibition/*Balanus amphitrite*	1.48 µM (EC_50_)	Seawater + DMSO	[16]
	Cytotoxicity	MTT/HCT-116	20.55 µM (IC_50_)	-	[16]
Kalihinol R (**122**)	Antifouling	Settlement inhibition/*Balanus amphitrite*	1.16 µM (EC_50_)	Seawater + DMSO	[16]
	Cytotoxicity	MTT/HCT-116	13.44 µM (IC_50_)	-	[16]
Kalihinol S (**123**)	Antifouling	Settlement inhibition/*Balanus amphitrite*	0.53 µM (EC_50_)	Seawater + DMSO	[16]
Kalihinol T (**124**)	Antifouling	Settlement inhibition/*Balanus amphitrite*	0.74 µM (EC_50_)	Seawater + DMSO	[16]
10-*epi*-Kalihinol X (**126**)	Cytotoxicity	MTT/A549	9.30 µg/mL (IC_50_)	-	[42]
	Antifouling	Settlement inhibition/*Balanus amphitrite*	0.69 µM (EC_50_)	Seawater + DMSO	[16]
	Cytotoxicity	MTT/HCT-116	8.21 µM (IC_50_)	-	[16]
Kalihiacyloxyamide C (**133**)	Cytotoxicity	SRB/L-02	14.6 μM (IC_50_)	Doxorubicin not detected	[62]
		MTT/K562	6.4 μM (IC_50_)	Doxorubicin ˂ 1.04 μM (IC_50_)	[62]
		SRB/ASPC-1	19.0 μM (IC_50_)	Doxorubicin ˂ 1.04 μM (IC_50_)	[62]
		SRB/H69AR	12.0 μM (IC_50_)	Doxorubicin 15.1 μM (IC_50_)	[62]
		SRB/MDA-MB-231	12.5 μM (IC_50_)	Doxorubicin ˂ 1.04 μM (IC_50_)	[62]
Kalihiacyloxyamide D (**134**)	Cytotoxicity	SRB/L-02	8.0 μM (IC_50_)	Doxorubicin not detected	[62]
		MTT/K562	6.3 μM (IC_50_)	Doxorubicin ˂ 1.04 μM (IC_50_)	[62]
		SRB/MDA-MB-231	7.3 μM (IC_50_)	Doxorubicin ˂ 1.04 μM (IC_50_)	[62]
Kalihiacyloxyamide G (**137**)	Cytotoxicity	SRB/L-02	19.2 μM (IC_50_)	Doxorubicin not detected	[62]
		MTT/K562	15.0 μM (IC_50_)	Doxorubicin ˂ 1.04 μM (IC_50_)	[62]
		SRB/MDA-MB-231	13.4 μM (IC_50_)	Doxorubicin ˂ 1.04 μM (IC_50_)	[62]
Kalihiacyloxyamide H (**138**)	Cytotoxicity	SRB/H69AR	16.8 μM (IC_50_)	Doxorubicin 15.1 μM (IC_50_)	[62]
		SRB/MDA-MB-231	12.5 μM (IC_50_)	Doxorubicin ˂ 1.04 μM (IC_50_)	[62]
Kalihinene (**139**)	Cytotoxicity	MTT/P388	1.2 μg/mL	-	[23]
	Antibacterial	Microbroth dilution/*B. subtilis* PY79	6.25 μg/mL (MIC)	-Trimethoprim 1.0 μg/mL (MIC) -Ciprofloxacin 1.0 μg/mL (MIC) -Rifampin 1.0 μg/mL (MIC) -Sulfamethoxazole 4.0 μg/mL (MIC) -Chloramphenicol 16.0 μg/mL (MIC) -Polymyxin B 16.0 μg/mL (MIC)	[21]
Kalihinene E (**145**)	Cytotoxicity	MTT/HCT-116	14.36 μM (IC_50_)	Camptothecin 9.25 μM (IC_50_)	[63]
		MTT/HeLa	13.36 μM (IC_50_)	Camptothecin 6.98 μM (IC_50_)	[63]
		MTT/QGY-7701	17.78 μM (IC_50_)	Camptothecin 4.05 μM (IC_50_)	[63]
		MTT/MDA-MB-231	12.84 μM (IC_50_)	Camptothecin 0.50 μM (IC_50_)	[63]
Kalihinene X (**147**)	Antifouling	Settlement inhibition/*Balanus amphitrite*	0.49 µg/mL (IC_50_)	CuSO_4_ 0.15 µg/mL (IC_50_)	[55,56]
	Antibacterial	Microbroth dilution/*B. subtilis* PY79	1.56 μg/mL (MIC)	-Trimethoprim 1.0 μg/mL (MIC) -Ciprofloxacin 1.0 μg/mL (MIC) -Rifampin 1.0 μg/mL (MIC) -Sulfamethoxazole 4.0 μg/mL (MIC) -Chloramphenicol 16.0 μg/mL (MIC) -Polymyxin B 16.0 μg/mL (MIC)	[21]
	Cytotoxicity	MTT/HCT-116	12.25 μM (IC_50_)	Camptothecin 9.25 μM (IC_50_)	[63]
		MTT/A549	8.55 μM (IC_50_)	Camptothecin 2.32 μM (IC_50_)	[63]
		MTT/HeLa	10.59 μM (IC_50_)	Camptothecin 6.98 μM (IC_50_)	[63]
		MTT/QGY-7701	13.02 μM (IC_50_)	Camptothecin 4.05 μM (IC_50_)	[63]
		MTT/MDA-MB-231	7.46 μM (IC_50_)	Camptothecin 0.50 μM (IC_50_)	[63]
Kalihinene Y (**148**)	Antifouling	Settlement inhibition/*Balanus amphitrite*	0.45 µg/mL (IC_50_)	CuSO_4_ 0.15 µg/mL (IC_50_)	[55,56]
	Antibacterial	Microbroth dilution/*B. subtilis* PY79	1.56 μg/mL (MIC)	-Trimethoprim 1.0 μg/mL (MIC) -Ciprofloxacin 1.0 μg/mL (MIC) -Rifampin 1.0 μg/mL (MIC) -Sulfamethoxazole 4.0 μg/mL (MIC) -Chloramphenicol 16.0 μg/mL (MIC) -Polymyxin B 16.0 μg/mL (MIC)	[21]
	Cytotoxicity	MTT/A549	16.12 μM (IC_50_)	Camptothecin 2.32 μM (IC_50_)	[63]
		MTT/HeLa	10.05 μM (IC_50_)	Camptothecin 6.98 μM (IC_50_)	[63]
		MTT/QGY-7701	14.41 μM (IC_50_)	Camptothecin 4.05 μM (IC_50_)	[63]
		MTT/MDA-MB-231	15.23 μM (IC_50_)	Camptothecin 0.50 μM (IC_50_)	[63]
Kalihinene Z (**149**)	Antifouling	Settlement inhibition/*Balanus amphitrite*	1.1 µg/mL (IC_50_)	CuSO_4_ 0.15 µg/mL (IC_50_)	[55,56]
10-Formamidokalihinene (**150**)	Antifouling	Settlement inhibition/*Balanus amphitrite*	0.095 µg/mL (IC_50_)	CuSO_4_ 0.15 µg/mL (IC_50_)	[55,56]
	Cytotoxicity	MTT/A549	6.98 μM (IC_50_)	Camptothecin 2.32 μM (IC_50_)	[63]
		MTT/HeLa	13.30 μM (IC_50_)	Camptothecin 6.98 μM (IC_50_)	[63]
		MTT/QGY-7701	14.53 μM (IC_50_)	Camptothecin 4.05 μM (IC_50_)	[63]
		MTT/MDA-MB-231	6.84 μM (IC_50_)	Camptothecin 0.50 μM (IC_50_)	[63]
15-Formamidokalihinene (**151**)	Antifouling	Settlement inhibition/*Balanus amphitrite*	0.14 µg/mL (IC_50_)	CuSO_4_ 0.15 µg/mL (IC_50_)	[55]
		MTT/A549	17.53 μM (IC_50_)	Camptothecin 2.32 μM (IC_50_)	[63]
		MTT/HeLa	14.74 μM (IC_50_)	Camptothecin 6.98 μM (IC_50_)	[63]
		MTT/QGY-7701	16.39 μM (IC_50_)	Camptothecin 4.05 μM (IC_50_)	[63]
Kalihipyran A (**165**)	Antifouling	Settlement inhibition/*Balanus amphitrite*	1.3 µg/mL (IC_50_)	CuSO_4_ 0.15 µg/mL (IC_50_)	[55]
	Cytotoxicity	MTT/A549	13.09 μM (IC_50_)	Camptothecin 2.32 μM (IC_50_)	[63]
		MTT/HeLa	11.19 μM (IC_50_)	Camptothecin 6.98 μM (IC_50_)	[63]
		MTT/QGY-7701	13.53 μM (IC_50_)	Camptothecin 4.05 μM (IC_50_)	[63]
Kalihipyran B (**166**)	Antifouling	Settlement inhibition/*Balanus amphitrite*	0.85 µg/mL (IC_50_)	CuSO_4_ 0.15 µg/mL (IC_50_)	[55]
Cavernene A (**169**)	Cytotoxicity	MTT/HCT-116	6.31 μM (IC_50_)	Camptothecin 9.25 μM (IC_50_)	[63]
	Cytotoxicity	MTT/HCT-116	8.99 μM (IC_50_)	Camptothecin 9.25 μM (IC_50_)	[63]
Biflora-4,9,15-triene (**168**)	Antifouling	Settlement inhibition/*Balanus amphitrite*	9.7 µg/mL (IC_50_)	CuSO_4_ 0.15 µg/mL (IC_50_)	[55]
Hymenialdisine (**185**)	Cytotoxicity	AlamarBlue/A2780S	146.8 μM (IC_50_)	Cisplatin 31.4 μM (IC_50_)	[82]
2β-Hydroxy-4,7-diketo-A-norcholest-5-en-2-oic acid (**208**)	Antifouling	Settlement inhibition/*Balanus albicostatus*	8.2 μg/mL EC_50_)	Capsaicin 1.32 μg/mL (EC_50_)	[74]
24*S*-Ethyl-2β-hydroxy-4,7-diketo-A-norcholest-5-en-2-oic acid (**209**)	Antifouling	Settlement inhibition/*Balanus albicostatus*	23.5 μg/mL (EC_50_)	Capsaicin 1.32 μg/mL (EC_50_)	[74]
2β-Hydroxy-4,7-diketo-24*R*-methyl-A-norcholest-5,22(*E*)-diene-2-oic acid (**210**)	Antifouling	Settlement inhibition/*Balanus albicostatus*	31.6 μg/mL (EC_50_)	Capsaicin 1.32 μg/mL (EC_50_)	[74]

Additionally, **100** displayed antimicrobial effectiveness versus *S. aureus*, *B. subtilis*, and *C. albicans* [83]. Compound **125** was found to have notable antimicrobial potential against *S. aureus*, *C. albicans*, and *T. mentagrophytes* [18]. Bugni et al. assessed the antibacterial activity of **88**, **100**, **104**–**108**, **116**, **125**, **127**, and **139** through in vitro inhibition of *B. subtilis* PY79 growth, as well as inhibition of bacterial folate biosynthesis using agar diffusion/microbroth dilution and luminescence rescue assays, respectively [21]. The results showed that the pyranyl-type **127** and **125** (MICs of 1.56 µg/mL) revealed a potent antibacterial potential; however, they only weakly inhibited the folate biosynthesis, suggesting an additional mechanism of action for these pyranyl derivatives. On the other hand, the furanyl-type **100**, **108**, and **139** displayed a more selective folate biosynthesis inhibition than pyranyl-type kalihinols. The existence of a formamido moiety at any position markedly decreased the activity, which could be due to a reduced cellular uptake. Additionally, the C-10 substitution pattern greatly affected the potency, which was evident by loss of activity in **88**, which differs in the C-10 isonitrile group orientation from the potent **127** and **125** (which have an *exo*-methylene and isothiocyanate groups, respectively) [21]. Xu et al. reported that **151** showed antifungal activity against *T. rubrum* and *M. gypseum* (MICs of 8.0 and 32.0 μg/mL, respectively), while **150** had activity against *C. albicans*, *C. neoformans*, *T. rubrum*, and *M. gypseum* (MICs of 4.0–8.0 μg/mL). It was noted that the isonitrile functionalities had a substantial role in antifungal activity [63]. The antifungal effects of **20**, **177**, **178**, **217**, and **220**–**222** against phytopathogenic fungi *Fusarium oxysporum* f. sp. *niveum*, *F. solani* f. sp. *cucurbitae*, *Pythium ultimum*, and *Alternaria solani* were assessed. Compounds **177**, **178**, and **220**–**222** had antifungal activities against *A. solani*, whereas **20** was the most active against *F. oxysporom*, *F. solani*, and *A. solani* (IZDs of 11.5 to 25.0 mm) [25]. None of them exhibited activity towards *P. ultimum* [25].

Fouling is the deposition and accumulation of living organisms (biofouling) and certain non-living materials on hard surfaces, most frequently in the aquatic environment, which results in serious economic problems [74]. Organotin compounds are the widely used chemicals for controlling these sessile organisms. These compounds are under criticism due to environmental and ecosystem concerns that necessitate the need for the development of nontoxic alternatives. Some soft corals and marine sponges produce environmentally friendly antifoulant secondary metabolites that provide chemical defenses against biofouling.

Compounds **5**, **9**, **23**, **32**, **66**, **75**, **76**, **85**, **89**, **91**, **92**, **98**, **99**, and **168** were examined for their antifouling potential through the prohibition of the settlement of the barnacle *Balanus amphitrite* larvae. Compounds **32**, **89**, **91**, **92**, **98**, and **99** inhibited metamorphosis and larval settlement by 100%. Additionally, **89**, **91**, **92**, **98**, and **99** showed 100% inhibition of the larval settlement and metamorphosis (at concentrations of 0.05 to 5.0 μg/mL). Isocyanate and isothiocyanate analogs **91** and **92** (EC_50_ ca. 0.05 μg/mL) had high antifouling capacities [40]. Compounds **65** and **67** (EC_50_s 0.50 and 0.53 µg/mL, respectively) prohibited *B. amphitrite* larval settlement with much less toxicity to larvae than CuSO_4_, while **65** had a much higher toxicity than **67** [49]. In addition, the related metabolites with isocyano and isothiocyanato moieties (**47**, **63**, and **64**) had marked antifouling capacities, with ED50 values of 7.2, 0.70, and 0.14 µg/mL, respectively [49]. In 2006, compounds **88**–**90** were evaluated against bacterial strains which induce *Hydroides elegans* larval settlement (*Micrococcus luteus*, *Loktanella hongkongensis*, *Pseudoalteromonas* sp., *Ruegeria CtaxMed-2*, *Rhodovulum* sp. MB-253, *Staphylococcus haemolyticus*, *Vibrio halioticoli*, *Vibrio* sp. NAP4, and *Vibrio fluvialis*) and marine pathogenic bacteria (*Shewanella algae*, *Moraxella phenylpyruvica*, *S. aureus*, *Vibrio vulnificus*, and *Vibrio furnissii*) using a disc diffusion assay [58]. These compounds strongly prohibited the growth of larval settlement-promoting strains *S. haemolyticus*, *L. hongkongensis*, and *M. luteus* (IZDs of 8.17–10.67 mm) compared to streptomycin. Additionally, they possessed powerful antibacterial efficacies versus pathogenic strains *V. furnissii* and *S. aureus* (IZDs of 5–7 mm) compared to streptomycin (IZD of 0.83–2.50 mm). Compound **88** was found to effectively suppress larval settlement (EC_50_ 0.5 μg/mL), and was further incorporated in Phytagel^®^. This compound slightly reduced thet bacterial abundance, but it modified the bacterial species composition in the biofilm, suggesting its capacity to change the bacterial community composition, which in turn modulates the larval settlement of fouling organisms [58]. Compounds **88**, **89**, **92, 98**, **115**, **119**–**124**, and **126** displayed significant antifouling activities against *B. amphitrite* larvae (EC_50_s of 0.41 to 1.43 μM) [16]. It was found that the compounds with an equatorial-Cl and isothiocyanate group had more activity. Compound **92** had a significant antifouling activity without cytotoxicity, which could indicate its potential as an antifouling agent [16]. Compounds **88**, **147**–**151**,**165**, **166**, and **168** were assessed for their antifouling activity against cyprid larvae of the barnacle *Balanus amphitrite*. The novel compounds **165** and **166** suppressed larval settlement and metamorphosis of the barnacle *Balanus amphitrite* (IC_50_s 1.3 and 0.85 µg/mL, respectively), which was comparable to **147**–**149** (IC_50_s of 0.49, 0.45, and 1.1 µg/mL, respectively) with low toxicity, whereas the isocyano derivatives **88**, **150**, and **151** (IC_50_s of 0.087, 0.095, and 0.14 µg/mL, respectively) were more efficient. Interestingly, **168** was moderately active (IC_50_ 4.6 µg/mL) and **88** and **150** were more active than CuSO_4_ (IC_50_ 0.15 µg/mL) [55,56]. Besides, **185** was found to have a significant antifouling capacity against *P. viridis* (EC_50_ 31.77 μg/mL in a mussel bioassay), *B. neritina* (EC_50_ 3.43 μg/mL in a bryozoan bioassay), and *U. prolifera* (EC_50_ 8.31 μg/mL in an algal bioassay). These results suggest that **185** may play a role in chemical defense against fouling in *Acanthella* sp. [69]. In addition to the potent anti-fouling diterpenes reported from *A. cavernosa*, Qui et al. stated that the nor-steroids **208**–**210** biosynthesized by *A. cavernosa* also demonstrated marked antifouling potentials (EC_50_s of 8.2, 23.5, and 31.6 μg/mL, respectively) towards the *Balanus albicostatus* larval settlement compared to capsaicin (EC_50_ of 1.32 μg/mL) in a settlement inhibition assay [74].

### 4.2. Cytotoxic Activity

The MeOH extracts of *A. elongata* from fishing nets of Kanyakumari revealed a toxicity (LC_50_ of 0.51 µg/mL) on *Artemia salina* in a brine shrimp bioassay [84]. In the MTT assay, *A. carteri* hydro-EtOH extracts had promising activities against HT-29, T47D, and Casky cell lines (IC_50_s of 37.56, 482.84, and 37.64 μg/mL, respectively) [85], while *A. acuta* MeOH extracts displayed notable cytotoxic activities versus LS174 and HeLa cell lines (IC_50_s of 9.92 and 29.51 μg/mL, respectively) [86].

Compounds **95** and **139** exhibited a cytotoxic efficacy on P388 murine leukemia cells (IC_50_s of 0.8 and 1.2 µg/mL, respectively) [61]. Compound **100** is a tetrahydrofuran-containing kalihinol with three isonitrile functionalities at C-5, C-10, and C-15 and was obtained from *Acanthella* sp. from the Cape Sada coast (Ehime Prefecture, Japan). This compound was found to have topoisomerase-I inhibition capacity by prohibiting the chromosome separation in *Asterina pectinifera* (fertilized starfish) eggs, while it did not affect topoisomerase II and DNA polymerases (α, β, and γ) [61]. In an MTT assay, **9, 17**, **97**, and **126** exhibited moderate in vitro cytotoxic capacities versus A549 (IC_50_s of 1.98–9.30 µg/mL) [42], whilst **88, 98**, **115, 119**–**121,** and **126** demonstrated cytotoxic potential against HCT-116 (IC_50_s of 5.97 to 28.67 μM) [16]. Compounds **169** and **170** showed potent cytotoxic potential against HCT-116 (IC_50_s of 6.31 and 8.99 μM, respectively) compared to camptothecin (IC_50_ of 9.25 μM), whereas **145** had activity against HCT-116, HeLa, QGY-7701, and MDA-MB-231 (IC_50_ of 12.84 to 17.78 μM) [63]. Furthermore, **157** was found to demonstrate a potent cytotoxic potential (IC_50_s of 6.57 and 3.60 µM, respectively) towards K562 and H69 cells, whereas **158** revealed moderate (IC_50_ of 8.73 µM) capacity against K562 cells compared to doxorubicin (IC_50_s of 0.252 and 0.980 µM, respectively) [64]. Compounds **133** and **135** exhibited cytotoxicity against K562 cell lines (IC_50_s of 6.4 and 6.3 μM, respectively), while **135** and **138** were active against MDA-MB-231 cell lines (IC_50_s of 7.3 and 7.9 μM, respectively) compared to doxorubicin (IC_50_ of ˂1 μM) [62]. Compounds **175**–**177** and **179** showed in vitro cytotoxic capacity against NSCLC-N6 (IC_50_s ranging from 4.8 to 11.2 μg/mL) [66]. In 2021, Abdullah et al. reported that **185** was less toxic versus A2780S cells (IC_50_ of 146.8 μM) and had no activity versus A2780CP compared to cisplatin (IC_50_ of 31.4 μM for A2780S and 76.9 μM for A2780CP) in an alamarBlue**^®^** assay [82]. The guanidine-containing alkaloids **191**–**193** were evaluated for the cellular stability of PDCD4 (Programmed cell death 4) using HEK-293 cells [24]. Compounds **191** and **193** were found to suppress TPA (tetradecanoylphorbol-13-acetate)-induced degradation of PDCD4 (EC_50_s of 1.8 and 2.8 μg/mL, respectively) compared to rapamycin (EC_50_ of 0.02 μg/mL), while **192** had no activity. It is noteworthy that **191** and **193** are the first marine natural metabolites that stabilized PDCD4 under tumor-promoting conditions [24].

### 4.3. Larvicidal and Antimalarial Activities

The larvicidal potential of *A. elongata* (doses of 0.01% to 10.0% for 24h) collected from Kanyakumari was assessed against s mosquito vector of filariasis, *Culex* sp. 3rd instar larvae. Its MeOH extract exhibited potent larvicidal capacity (LC_50_ of 0.066 mg/mL), suggesting the potential of *A. elongata* extracts for the development of novel larvicidal agents [87]. Whilst the *n*-BuOH, *n*-hexane, aqueous, and EtOAc fractions (IC50s of 9.5, 4.6, 15.0, and 3.3 µg/mL, respectively) of *A. cavernosa* extracts had the ability to prohibit heme polymerization, revealing their anti-malarial potential [88].

Compounds **17**, **23**, **42**, and **45** demonstrated in vitro, dose-dependent antimalarial activity against *P. falciparum (*IC_50_s of 142 to 12340 ng/mL for D6 and 16.5 to 3110 ng/mL for W2) compared to chloroquine (IC_50_ of 1.95 ng/mL for D6 and 22.8 ng/mL for W2), where **17** was the most active. It was found that the isonitrile group was crucial and isothiocyanate derivative **23** was 500-fold less active than **17**. The eudesmane isothiocyanates **42** and **45** have lower potential than **17** [19]. Compounds **88**, **108**, **110**, **111**, **114**, **115**, **127**, **129**, **139**, and **153** were assessed for their antimalarial activity [57]. Among the tested compounds, **88** was found to display potent in vitro (EC_50_ of 1.2 × 10^−9^ M) and selective (SI of 317) antimalarial effectiveness, whereas **115**, **110**, **139**, and **153** had EC_50_s from 8.0 × 10^−8^ M to ˃ 1.8 × 10^−6^ M against *P*. *falciparum* compared to mefloquine (EC_50_ of 3.2 × 10^−8^ M) [57].

### 4.4. Cyclooxygenase Inhibitory and α2B Adrenoceptor Agonistic Activities

Compound **84** displayed promising dose-dependent anti-inflammatory activity through inhibition of TNF-α and CCL2 (% inhibition ratios of 74.1% and 64.1%, respectively) at a concentration of 1 μM in lipopolysaccharide (LPS)-stimulated RAW 264.7 macrophages [43]. Yan et al. reported that **88** revealed significant COX-2 (IC_50_ of 1.07 µM) inhibitory activity [41]. In 2009, Davis et al. stated that **188** showed α2B adrenoceptor agonist activity (EC_50_ of 4.2 μM) compared to noradrenaline in a α2B adrenoceptor FLIPR (fluorescence imaging plate reader) assay in HEK 293 cells [70].

### 4.5. Insecticidal and Anthelmintic Activities

Hammami et al. evaluated the insecticidal effect of **34** against *Tribolium confusum* Duv (major pest of stored products) using the direct contact application method; it was found to prohibit *Tribolium confusum* Duv larvae growth (45% mortality at a concentration of 10 mg/mL) [25]. An in vitro anthelmintic assay of *A. carvenosa*-derived kalihinols against *Nippostrongylus brasilliensis* (gastrointestinal roundworm) (at 50 µg/mL) demonstrated that **127** had extreme activity; however, **114**, **88**, **125**, and **130** were potent and **101** was inactive [18,53].

### 4.6. Antioxidant Activity

*A. carteri* hydro-EtOH extracts revealed a DPPH scavenging ability (IC_50_ of 56.94 μg/mL) compared to ascorbic acid (IC_50_ of 0.67 μg/mL) [85]. Putra et al. stated that *n-*BuOH fractions of *A. cavernosa* had the largest phenolic content, followed by EtOAc, aqueous, and *n-*hexane fractions. These fractions demonstrated antioxidant potential with the % inhibition of DPPH radicals ranging from 16.40 to 40.57%, whereas the *n-*hexane fraction displayed the most powerful DPPH radical suppression (at a concentration of 171.86 µg/mL) [88].

## 5. Nanoparticles

Synthesis of nanoparticles (NPs) with green technology is beneficial over chemical procedures because of their lower environmental impacts [89,90]. The use of biological extracts of living organisms, such as actinomycetes, bacteria, yeast, plants, marine sponges, and fungi, in green synthetic processes indicates their considerable potential for NP synthesis [89]. Some researchers have reported the biosynthesis of NPs using species of the genus *Acanthella* that are cost-effective and compatible with pharmaceutical and biomedical applications and could be utilized commercially for large-scale production. In 2010, Inbakandan et al. synthesized highly stable AuNPs (gold nanoparticles) using an *A. elongata* extract by reducing aqueous AuCl_4_^−^, suggesting that this sponge is a perfect candidate for AuNP synthesis [91]. In another study in 2012, AgNPs were synthesized using the H_2_O-soluble extract of *A. elongata*. These NPs were characterized by UV, XRD (X-ray diffraction), TEM (transmission electron microscopy), and FTIR (Fourier transform infrared spectroscopy). It was found that amines of the sponge extract were accountable for the bio-reduction of the silver salt to the AgNps [90].

## 6. Conclusions

**Figure 21:**
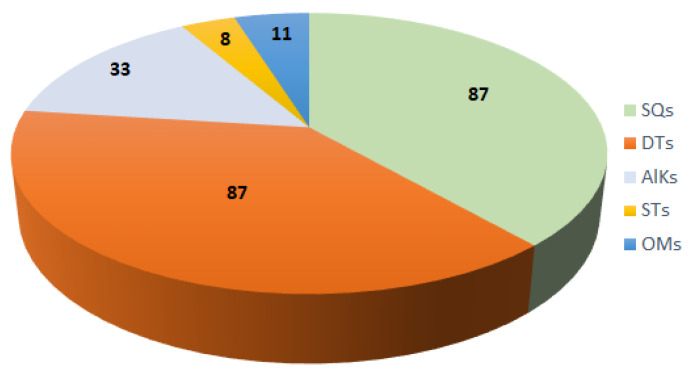
Different classes of metabolites reported from the genus *Acanthella*. SQs: sesquiterpenes; DTs: diterpenes; AlKs: alkaloids; STs: steroid compounds; OMs: other metabolites.

The current work presents extensive documentation of the reported studies on the genus *Acanthella* with a special focus on their diverse chemical classes of metabolites and their bioactivities. The sponges of this genus were obtained from various marine environments. A total of 226 metabolites from various species of this genus were reported in the period from 1974 to the beginning of 2023. These metabolites illustrated in this work belong mainly to terpene (sesqui- and di-terpenes), alkaloid, and steroid chemical classes (Figure 21).

**Figure 22:**
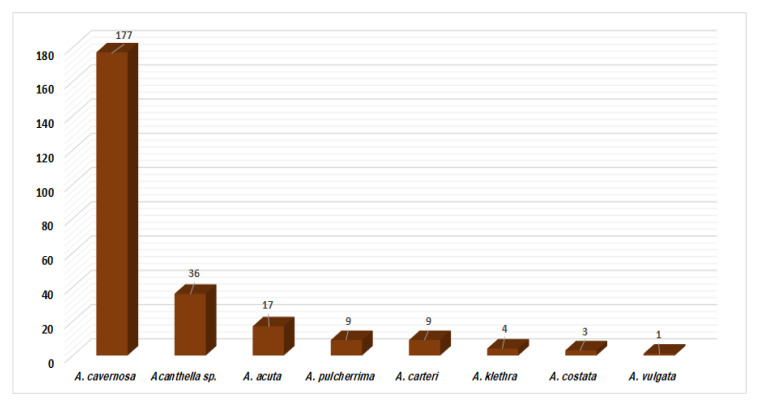
Number of compounds reported from each studied *Acanthella* species.

Metabolites have been reported from *A. cavernosa*, *A. pulcherrima*, *A. klethra*, *A. acuta*, *A. carteri*, *A. costata*, *A. vulgate*, and unidentified *Acanthella* species. *A. cavernosa* (177 compounds), *Acanthella* sp. (36 compounds), and *A. acuta* (17 compounds) are frequently studied members of this genus in terms of the number of isolated compounds and have proven to be rich in terpenes and alkaloids (Figure 22). Interestingly, kalihinane-type diterpenoids are commonly purified from *A. carvenosa*, which could be a chemotaxonomic marker for this sponge.

In addition, bioactivity investigations of some species extracts are detailed in the literature. Sesquiterpene- and diterpene-containing nitrogen and alkaloids were the dominant metabolites reported from the species of genus *Acanthella*. These metabolites were mainly assessed for antifouling, antimicrobial, and cytotoxic activities. Limited studies have reported the larvicidal, antimalarial, cyclooxygenase inhibitory, α2B adrenoceptor agonistic, insecticidal, and anthelmintic capacities of these compounds. It is noteworthy that some kalihinol and kalihinene diterpenes have marked antifouling potentials. Additionally, **88** demonstrated notable antifouling, cytotoxic, antimalarial, COX-2 inhibition, and anthelmintic activities. The diverse structural features and bioactivities demonstrated by some of these metabolites make them attractive biological targets that are worthy of further investigation.

## Figures and Tables

**Table 1:** List of the accepted species of the genus *Acanthella* and their status [14].

Species	Accepted as	Type Locality
*Acanthella aculeata* (Thiele, 1898)	-	Central Kuroshio Japan
*Acanthella annulata* (Sarà, 1958)	-	Western Mediterranean ^#^
*Acanthella acuta* (Schmidt, 1862)	-	Adriatic Sea ^#^
*Acanthella aurantiaca* (Keller, 1889) *	*Stylissa carteri* (Dendy, 1889)	Southern Red Sea
*Acanthella branchia* Sim (Kim and Byeon, 1990)		East China Sea South Korea
*Acanthella cactiformis* (Carter, 1885) **	*Rhaphoxya cactiformis* (Carter, 1885)	Bassian
*Acanthella calyx* (Dendy, 1922)		Cargados Carajos/Tromelin Island ^#^
*Acanthella carduus* (Lamarck, 1814) **	*Phakellia carduus* (Lamarck, 1814)	-
*Acanthella carteri* (Dendy, 1889)	*Stylissa carteri* (Dendy, 1889)	South India Sri Lanka
*Acanthella cavernosa* (Dendy, 1922)	-	Seychelles ^#^
*Acanthella columnata* (Burton, 1928)	*Phakellia columnata* (Burton, 1928)	-
*Acanthella corrugata* (George and Wilson, 1919)	*Axinella corrugata* (George and Wilson, 1919)	Virginian
*Acanthella costata* (Kieschnick, 1898)		Banda Sea Ambon
*Acanthella cristagalli* (Dendy, 1924) **	*Tedania (Tedaniopsis) cristagalli* Dendy, 1924)	-
*Acanthella cubensis* (Alcolado, 1984)	-	Greater Antilles
*Acanthella danerii* Costa (Bavestrello, Pansini, and Bertolino, 2020)	-	Chilean Exclusive Economic Zone ^#^ Chiloense
*Acanthella dendyi* (Bergquist, 1970)	-	Northeastern New Zealand
*Acanthella ehrenbergi* (Keller, 1889) **	*Biemna ehrenbergi* (Keller, 1889)	Southern Red Sea
*Acanthella elongata* (Dendy, 1905)	*Auletta elongata* (Dendy, 1905)	-
*Acanthella erecta* (Carter, 1876)	-	Celtic Seas
*Acanthella euctimena* (Hentschel, 1912) **	*Phakettia euctimena* (Hentschel, 1912)	Arafura Sea
*Acanthella flabellata* (Tanita, 1961)	-	Central Kuroshio Current
*Acanthella flabelliformis* (Keller, 1889) **	*Echinodictyum flabelliforme* (Keller, 1889)	Southern Red Sea
*Acanthella flagelliformis* (van Soest and Stentoft, 1988)		Eastern Caribbean ^#^
*Acanthella gorgonoides* (Thomas, 1984)	-	Eastern India
*Acanthella hirciniopsis* (Carter, 1885) *	*Rhaphoxya cactiformis* (Carter, 1885)	Bassian
*Acanthella hispida* (Pulitzer-Finali, 1982)		Southern China ^#^
*Acanthella inflexa* (Pulitzer-Finali, 1982) **	*Stylissa inflexa* (Pulitzer-Finali, 1982)	Central and Southern Great Barrier Reef ^#^
*Acanthella insignis* (Thiele, 1898)	-	Central Kuroshio Current Japan
*Acanthella klethra* (Pulitzer-Finali, 1982)	-	Central and Southern Great Barrier Reef ^#^
*Acanthella ligulata* (Burton, 1928)	-	Andaman Sea Coral Coast
*Acanthella lyrata* (Esper, 1794) **	*Auletta lyrata* (Esper, 1794)	-
*Acanthella mastophora* (Schmidt, 1870)	-	Floridian ^#^
*Acanthella megaspicula* (Thomas, 1984)	-	Eastern India
*Acanthella minuta* (Tanita, 1968)	-	Central Kuroshio Current
*Acanthella multiformis* (Vosmaer, 1885)	-	Northern Norway and Finnmark
*Acanthella obtusa* (Schmidt, 1862) **	*Dictyonella obtusa* (Schmidt, 1862)	Adriatic Sea
*Acanthella oviforma* (Tanita and Hoshino, 1989)	-	Central Kuroshio Current
*Acanthella pulcherrima* (Ridley and Dendy, 1886)	-	Central Kuroshio Current
*Acanthella ramosa* (Kumar, 1925)	-	Eastern India ^#^
*Acanthella ramus* (Sim, Kim, and Byeon, 1990)	-	East China Sea ^#^
*Acanthella saladinorum* (Sim-Smith, Hickman Jr, and Kelly, 2021)	-	Eastern Galapagos Islands ^#^
*Acanthella simplex* (Thiele, 1898)	-	Central Kuroshio Current
*Acanthella stanleei* (Nascimento, Cavalcanti, and Pinheiro, 2019)	-	Northeastern Brazil ^#^
*Acanthella stipitata* (Carter, 1881) **	*Phakellia stipitata* (Carter, 1881)	Bassian
*Acanthella styloida* (Tanita and Hoshino, 1989)	-	Central Kuroshio Current
*Acanthella tenuispiculata* (Dendy, 1897)	-	Great Australian Bight, Australia
*Acanthella vaceleti* (van Soest and Stentoft, 1988)	-	Eastern Caribbean ^#^ Barbados Exclusive Economic Zone
*Acanthella vulgata* (Thiele, 1898)	-	Central Kuroshio Current
*Acanthella xutha* (de Laubenfels, 1954)	-	East Caroline Islands

* Genus transfer and junior synonym; ** genus transfer; ^#^ halotype.

**Table 2:** Sesquiterpenes from the genus *Acanthella* (molecular weight and formulae, chemical class, species, and sampling locations).

Compound Name	Mol. Wt.	Mol. Formula	Species	Sampling Locations	Ref.
Aromadendrane-type sesquiterpenes					
Axisonitrile-2 (**1**)	231	C_16_H_25_N	*A. cavernosa*	Hachijo-Jima Island, Japan	[32]
(+)-Axamide 2 (**2**)	249	C_16_H_27_NO	*A. cavernosa*	Coast of Ximao Island, Hainan, China	[33]
l-Isocyanoaromadendrane (**3**)	231	C_16_H_25_N	*A. acuta*	Near Banyuls, France	[34,35]
	-	-	*A. pulcherrima*	Weed Reef, Darwin, Australia	[35]
	-	-	*A. acuta*	Near Banyuls, France	[36]
	-	-	*A. cavernosa*	Tani’s Reef, Mooloolaba, Australia	[37]
	-	-	*A. cavernosa*	Tani’s Reef or Coral Gardens dive sites	[38]
Isonitrile 2 (**4**)	231	C_16_H_25_N	*A. acuta*	Bay of Naples, southern Italy	[39]
(+)-Axisothiocyanate 2 (**5**)	263	C_16_H_25_NS	*A. cavernosa*	Hachijo-jima Island, Japan	[40]
	-	-	*Acanthella* sp.	Ximao Sea, Hainan, China	[41]
	-	-	*A. cavernosa*	Tani’s Reef or Coral Gardens dive sites	[38]
l-Isothiocyanatearomadendrane (**6**)	263	C_16_H_25_NS	*A. acuta*	Near Banyuls, France	[36]
	-	-	*A. cavernosa*	Tani’s Reef, Mooloolaba, Australia	[37]
1-Isocyanatearomadendrane (**7**)	247	C_16_H_25_NO	*A. acuta*	Near Banyuls, France	[36]
	-	-	*A. cavernosa*	Tani’s Reef or Coral Gardens dive sites	[38]
1-Isothiocyanatoaromadendrane (**8**)	263	C_16_H_25_NS	*A. acuta*	Bay of Naples, southern Italy	[39]
	-	-	*A. cavernosa*	Tani’s Reef or Coral Gardens dive sites	[38]
(+)-10*R*-Isothiocyanatoalloaromadendrane (**9**)	263	C_16_H_25_NS	*A. cavernosa*	Hachijo-jima Island, Japan	[40]
	-	-	*Acanthella* sp.	Ximao Sea, Hainan, China	[41]
	-	-	*Acanthella* sp.	Yalong Bay, Hainan, China	[42]
	-	-	*A. cavernosa*	Coast of Ximao Island, Hainan, China	[33]
10α-Isothiocyanoalloaromadendrane (**10**)	263	C_16_H_25_NS	*A. pulcherrima*	Weed Reef, Darwin, Australia	[35]
	-	-	*A. cavernosa*	Tani’s Reef or Coral Gardens dive sites	[38]
	-	-	*Acanthella* sp.	Ximao Sea, Hainan, China	[41]
	-	-	*A. cavernosa*	Tani’s Reef, Mooloolaba, Australia	[37]
Ximaocavernosin O (**11**)	368	C_24_H_36_N_2_O	*A. cavernosa*	Coast of Ximao Island, Hainan, China	[33]
*ent*-4β,10α-Dihydroxyaromadendrane (**12**)	238	C_15_H_26_O_2_	*A. cavernosa*	Coast of Ximao Island, Hainan, China	[43]
Palustrol (**13**)	222	C_15_H_26_O	*A. acuta*	Near Banyuls, France	[34]
	-	-	*A. acuta*	Near Banyuls, France	[36]
10*S*-Viridiflorol (**14**)	222	C_15_H_26_O	*A. cavernosa*	Hachijo-jima Island, Japan	[40]
10*R*-Viridiflorol (**15**)	222	C_15_H_26_O	*A. cavernosa*	Hachijo-jima Island, Japan	[40]
(+)-Ximaocavernosin P (**16**)	234	C_15_H_22_O_2_	*A. cavernosa*	Coast of Ximao Island, Hainan, China	[43]
Spiroaxane-type sesquiterpenes					
Axisonitrile-3 (**17**)	231	C_16_H_25_N	*A. acuta*	Near Banyuls, France	[34]
	-	-	*A. cavernosa*	Thailand	[18]
	-	-	*A. klethra*	Pelorus Island, Queensland, Australia	[19]
	-	-	*A. klethra*	Vicinities of Phantom and Pelom Islands, Queensland, Australia	[44]
	-	-	*A. cavernosa*	Heron Island, Great Barrier Reef, Australia	[31]
	-	-	*A. cavernosa*	Heron Island, Great Barrier Reef, Australia	[45]
	-	-	*Acanthella* sp.	Ximao Sea, Hainan, China	[41]
	-	-	*A. cavernosa*	Tani’s Reef, Mooloolaba, Australia	[37]
Isonitrile 1 (**18**)	245	C_17_H_27_N	*A. acuta*	Bay of Naples, southern Italy	[39]
3-Oxoaxisonitrile-3 (**19**)	245	C_16_H_23_NO	*Acanthella* sp.	Ximao Sea, Hainan, China	[41]
Axisonitrile-4 (**20**)	231	C_16_H_25_N	*A. acuta*	Sidi Elghdamssi island, Monastir region, Tunisia	[25]
Axisocyanate-3 (**21**)	247	C_16_H_25_NO	*A. cavernosa*	Tani’s Reef, Mooloolaba, Australia	[37]
(+)-Axisothiocyanate (**22**)	263	C_16_H_25_NS	*A. cavernosa*	Coast of Ximao Island, Hainan, China	[33]
Axisothiocyanate 3 (**23**)	263	C_16_H_25_NS	*A. klethra*	Pelorus Island, Queensland, Australia	[19]
	-	-	*A. klethra*	Vicinities of Phantom and Pelom Islands, Queensland, Australia	[44]
	-	-	*Acanthella* sp.	Queen Charlotte Island chain off the coast of British Columbia.	[46]
	-	-	*A. cavernosa*	Hachijo-jima Island, Japan	[40]
	-	-	*A. cavernosa*	Heron Island, Great Barrier Reef, Australia	[31]
	-	-	*A. cavernosa*	Heron Island, Great Barrier Reef, Australia	[45]
Ximaocavernosin H (**24**)	277	C_16_H_22_NOS	*A. cavernosa*	Coast of Ximao Island, Hainan, China	[33]
Ximaocavernosin I (**25**)	279	C_16_H_25_NOS	*A. cavernosa*	Coast of Ximao Island, Hainan, China	[33]
Ximaocavernosin J (**26**)	279	C_16_H_25_NOS	*A. cavernosa*	Coast of Ximao Island, Hainan, China	[33]
Ximaocavernosin K (**27**)	263	C_16_H_25_NO_2_	*A. cavernosa*	Coast of Ximao Island, Hainan, China	[33]
Ximaocavernosin L (**28**)	265	C_16_H_27_NO_2_	*A. cavernosa*	Coast of Ximao Island, Hainan, China	[33]
Ximaocavernosin M (**29**)	265	C_16_H_27_NO_2_	*A. cavernosa*	Coast of Ximao Island, Hainan, China	[33]
Ximaocavernosin N (**30**)	368	C_24_H_36_N_2_O	*A. cavernosa*	Coast of Ximao Island, Hainan, China	[33]
(–)-Axamide 3 (**31**)	249	C_16_H_27_NO	*A. cavernosa*	Coast of Ximao Island, Hainan, China	[33]
Axamide 3 (**32**)	249	C_16_H_27_NO	*A. cavernosa*	Hachijo-jima Island, Japan	[40]
Isothiocyanate 1 (**33**)	277	C_17_H_27_NS	*A. acuta*	Bay of Naples, southern Italy	[39]
Eudesmane-type sesquiterpenes					
Acanthellin-1 (**34**)	231	C_16_H_25_N	*A. acuta*	Bay of Naples, southern Italy	[30]
	-	-	*A. acuta*	Near Banyuls, France	[34]
	-	-	*A. acuta*	Bay of Taranto near Porto Cesareo, Southern Italy	[47]
	-	-	*A. acuta*	Sidi Elghdamssi Island, Monastir region, Tunisia	[25]
Acanthene B (**35**)	263	C_16_H_25_NS	*Acanthella* sp.	Queen Charlotte Island chain off the coast of British Columbia.	[46]
	-	-	*Acanthella* sp.	Ximao Sea, Hainan, China	[41]
	-	-	*A. cavernosa*	Tani’s Reef or Coral Gardens dive sites	[38]
Acanthine B (**36**)	263	C_16_H_25_NS	*A. cavernosa*	Coast of Ximao Island, Hainan, China	[33]
Acanthene C (**37**)	249	C_16_H_27_NO	*Acanthella* sp.	Queen Charlotte Island chain off the coast of British Columbia.	[46]
Axiriabiline A (**38**)	249	C_16_H_27_NO	*A. cavernosa*	Xidao Island, Hainan, China	[48]
6α-Isocyano-5αH,7αH,10α-eudesm-4(14)-ene) (**39**)	231	C_16_H_25_N	*A. acuta*	Bay of Taranto near Porto Cesareo, Southern Italy	[47]
	-	-	*A. acuta*	Bay of Naples, southern Italy	[39]
11-Isocyano-7βH-eudesm-5-ene (**40**)	231	C_16_H_25_N	*Acanthella* sp.	Queen Charlotte Island chain off the coast of British Columbia.	[46]
11-Formamido-7βH-eudesm-5-ene (**41**)	249	C_16_H_27_NO	*A. cavernosa*	Xidao Island, Hainan, China	[48]
11-Isothiocyano-7βH-eudesm-5-ene (**42**)	263	C_16_H_25_NS	*A. pulcherrima*	Weed Reef, Darwin, Australia	[35]
	-	-	*A. klethra*	Pelorus Island, Queensland, Australia	[19]
	-	-	*A. klethra*	Vicinities of Phantom and Pelom Islands, Queensland, Australia	[44]
	-	-	*Acanthella* sp.	Queen Charlotte Island chain off the coast of British Columbia.	[46]
	-	-	*A. cavernosa*	Heron Island, Great Barrier Reef, Australia	[31]
6α-Isothiocyano-5αH,7αH,10α-eudesm-4(14)-ene (**43**)	263	C_16_H_25_NS	*A. acuta*	Bay of Taranto near Porto Cesareo, Southern Italy	[47]
	-	-	*A. acuta*	Bay of Naples, southern Italy	[39]
	-	-	*Acanthella* sp.	Queen Charlotte Island chain off the coast of British Columbia.	[46]
(l*R*,5*R*,6*R*,8*R*)-Dec[4.4.0]ane-1,5-dimethyl-8-(1′-methylethenyl)-5-isothiocyanate (**44**)	263	C_16_H_25_NS	*A. klethra*	Pelorus Island, Queensland, Australia	[19]
	-	-	*A. klethra*	Vicinities of Phantom and Pelom Islands, Queensland, Australia	[44]
	-	-	*Acanthella* sp.	Queen Charlotte Island chain off the coast of British Columbia.	[46]
(l*R*,5*R*,6*R*,8*S*)-Dec[4.4.0]ane-1,5-dimethyl-8-(1′-methylethenyl)-5-isothiocyanate (**45**)	263	C_16_H_25_NS	*A. klethra*	Pelorus Island, Queensland, Australia	[19]
	-	-	*A. klethra*	Vicinities of Phantom and Pelom Islands, Queensland, Australia	[44]
	-	-	*A. cavernosa*	Heron Island, Great Barrier Reef, Australia	[45]
Cadinene-type sesquiterpenes					
10-Isothiocyanato-4-cadinene (**46**)	263	C_16_H_25_NS	*A. cavernosa*	Heron Island, Great Barrier Reef, Australia	[45]
	-	-	*A. cavernosa*	Tani’s Reef or Coral Gardens dive sites	[38]
10-Isothiocyanato-4-amorphene (**47**)	263	C_16_H_25_NS	*A. cavernosa*	Heron Island, Great Barrier Reef, Australia	[45]
	-	-	*A. cavernosa*	Several locations off the Japanese coast	[49]
	-	-	*Acanthella* sp.	Ximao Sea, Hainan, China	[41]
	-	-	*A. cavernosa*	Tani’s Reef or Coral Gardens dive sites	[38]
Isomer-10-isothiocyanato-4-amorphene (**48**)	263	C_16_H_25_NS	*A. cavernosa*	Heron Island, Great Barrier Reef, Australia	[45]
(+)-Ximaocavernosin A (**49**)	295	C_16_H_25_NO_2_S	*A. cavernosa*	Coast of Ximao Island, Hainan, China	[43]
(±)-Ximaocavernosin A (**50**)	279	C_16_H_25_NOS	*A. cavernosa*	Coast of Ximao Island, Hainan, China	[33]
(±)-Ximaocavernosin B (**51**)	279	C_16_H_25_NOS	*A. cavernosa*	Coast of Ximao Island, Hainan, China	[33]
(±)-Ximaocavernosin C (**52**)	279	C_16_H_25_NOS	*A. cavernosa*	Coast of Ximao Island, Hainan, China	[33]
(+)-Ximaocavernosin D (**53**)	293	C_17_H_27_NOS	*A. cavernosa*	Coast of Ximao Island, Hainan, China	[33]
(±)-Ximaocavernosin E (**54**)	295	C_16_H_25_NO_2_S	*A. cavernosa*	Coast of Ximao Island, Hainan, China	[33]
(+)-Ximaocavernosin F (**55**)	309	C_17_H_27_NO_2_S	*A. cavernosa*	Coast of Ximao Island, Hainan, China	[33]
(±)-Ximaocavernosin G (**56**)	277	C_16_H_23_NOS	*A. cavernosa*	Coast of Ximao Island, Hainan, China	[33]
(±)-Axinisothiocyanate J (**57**)	279	C_16_H_25_NOS	*A. cavernosa*	Coast of Ximao Island, Hainan, China	[33]
(±)-Axinisothiocyanate D (**58**)	295	C_16_H_25_NO_2_S	*A. cavernosa*	Coast of Ximao Island, Hainan, China	[33]
Axinisothiocyanate A (**59**)	295	C_16_H_25_NO_2_S	*A. cavernosa*	Coast of Ximao Island, Hainan, China	[33]
ent-Epicubenol (**60**)	222	C_15_H_26_O	*A. pulcherrima*	Weed Reef, Darwin, Australia	[35]
Isothiocyanate 4 (**61**)	263	C_16_H_25_NS	*A. pulcherrima*	Weed Reef, Darwin, Australia	[35]
Epipolasin-A enantiomer-2 (**62**)	263	C_16_H_25_NS	*A. pulcherrima*	Weed Reef, Darwin, Australia	[35]
10α-Isocyano-4-amorphene (**63**)	231	C_16_H_25_N	*A. cavernosa*	Hachijo-Jima Island, Japan	[32]
	-	-	*A. cavernosa*	Several locations off the Japanese coast	[49]
10-Isocyano-4-cadinene (**64**)	231	C_16_H_25_N	*A. cavernosa*	Heron Island, Great Barrier Reef, Australia	[45]
	-	-	*A. cavernosa*	Several locations off the Japanese coast	[49]
	-	-	*A. cavernosa*	Tani’s Reef or Coral Gardens dive sites	[38]
10-Formamido-4-cadinene (**65**)	249	C_16_H_27_NO	*A. cavernosa*	Several locations off the Japanese coast	[49]
	-	-	*A. cavernosa*	Xidao Island, Hainan, China	[48]
(+)-α-Muurolene (**66**)	204	C_15_H_24_	*A. cavernosa*	Hachijo-jima Island, Japan	[40]
T-cadinol (**67**)	222	C_15_H_26_O	*A. cavernosa*	Several locations off the Japanese coast	[49]
(+)-Maninsigin D (**68**)	234	C_15_H_22_O_2_	*A. cavernosa*	Coast of Ximao Island, Hainan, China	[43]
	234	C_15_H_22_O_2_	*A. cavernosa*	Coast of Ximao Island, Hainan, China	[43]
(-)-Maninsigin D (**69**)	234	C_15_H_22_O_2_	*A. cavernosa*	Coast of Ximao Island, Hainan, China	[43]
(+)-Ximaocavernosin Q (**70**)	234	C_15_H_22_O_2_	*A. cavernosa*	Coast of Ximao Island, Hainan, China	[43]
(-)-Ximaocavernosin Q (**71**)	234	C_15_H_22_O_2_	*A. cavernosa*	Coast of Ximao Island, Hainan, China	[43]
Cadalene (**72**)	198	C_15_H_18_	*A. cavernosa*	Coast of Ximao Island, Hainan, China	[43]
*trans*-4,5-Dihydroxycorocalane (**73**)	234	C_15_H_22_O_2_	*A. cavernosa*	Coast of Ximao Island, Hainan, China	[43]
Axane-type sesquiterpenes					
Cavernoisonitrile (**74**)	245	C_16_H_23_NO	*A. cavernosa*	Hachijo-Jima Island, Japan	[32]
(-)-Cavernothiocyanate (**75**)	263	C_16_H_25_NS	*A. cavernosa*	Hachijo-Jima Island, Japan	[32]
	-	-	*A. cavernosa*	Hachijo-jima Island, Japan	[40]
1β-H,7α-methyl,8α-H,9β-methyl-Cavernothiocyanate (**76**)	263	C_16_H_25_NS	*A. cavernosa*	Hachijo-jima Island, Japan	[40]
Bisabolene-type sesquiterpenes					
7-Isocyano-7,8-dihydro-α-bisabolene (**77**)	231	C_16_H_25_N	*A. cavernosa*	Hachijo-Jima Island, Japan	[32]
Epimaaliane-type sesquiterpenes					
(+)-Epipolasin-A (**78**)	263	C_16_H_25_NS	*A. pulcherrima*	Weed Reef, Darwin, Australia	[35]
	-	-	*A. cavernosa*	Heron Island, Great Barrier Reef, Australia	[31]
Epipolasin-A enantiomer-1 (**79**)	263	C_16_H_25_NS	*A. pulcherrima*	Weed Reef, Darwin, Australia	[35]
	-	-	*Acanthella* sp.	Ximao Sea, Hainan, China	[41]
5-Formamide-isonitrile (**80**)	249	C_16_H_27_NO	*Acanthella* sp.	Queen Charlotte Island chain off the coast of British Columbia.	[46]
Isonitrile 4 (**81**)	231	C_16_H_25_N	*Acanthella* sp.	Queen Charlotte Island chain off the coast of British Columbia.	[46]
Maaliol (**82**)	222	C_15_H_26_O	*A. pulcherrima*	Weed Reef, Darwin, Australia	[35]
Acanthene A (**83**)	240	C_15_H_25_Cl	*Acanthella* sp.	Queen Charlotte Island chain off the coast of British Columbia.	[46]
Gurjunene					
(+)-Aristolone (**84**)	218	C_15_H_22_O	*A. cavernosa*	Coast of Ximao Island, Hainan, China	[43]
	-	-	*A. cavernosa*	South China Sea	[50]
(+)-9-Aristolene (**85**)	204	C_15_H_24_	*A. cavernosa*	Hachijo-jima Island, Japan	[40]
Isonitrile 3 (**86**)	231	C_16_H_25_N	*A. acuta*	Bay of Naples, southern Italy	[39]
Isothiocyanate 3 (**87**)	263	C_16_H_25_NS	*A. acuta*	Bay of Naples, southern Italy	[39]

**Table 3:** Diterpenes from the genus *Acanthella* (molecular weight and formulae, chemical class, species, and sampling locations).

Compound Name	Mol. Wt.	Mol. Formula	Species	Sampling Locations	Ref.
Kalihinols					
Kalihinol A (**88**)	392	C_22_H_33_ClN_2_O_2_	*Acanthella* sp.	Apra Harbor, Guam, western side of the USA	[52]
	-	-	*Acanthella* sp.	Apra Harbor, Guam, western side of the USA	[22]
	-	-	*A. caruenosa*	Fiji, South Pacific Ocean	[53]
	-	-	*A. klethra*	Kuchinoerabu Island of the Satsunan Archipelago, Japan	[23]
	-	-	*A. cavernosa*	Seychelles and Desnceufs Islands	[54]
	-	-	*A. cavernosa*	Fiji, South Pacific Ocean	[51]
	-	-	*A. cavernosa*	Yakushima Island, southwest of Tokyo	[55,56]
	-	-	*Acanthella* sp.	Coral reef of Ishigaki Island, Okinawa, Japa	[57]
	-	-	*A. cavernosa*	Dibud, Philippines	[21]
	-	-	*A. cavernosa*	Shallow water reef in Sanya Bay, Hainan Island, China	[58]
	-	-	*Acanthella* sp.	Ximao Sea, Hainan, China	[41]
	-	-	*Acanthella* sp.	Yalong Bay, Hainan, China	[42]
	-	-	*A. cavernosa*	Xisha Islets, South China Sea	[16]
	-	-	*A. cavernosa*	South China Sea	[50]
*trans* 10β-Formamidokalihinol A (**89**)	410	C_22_H_35_ClN_2_O_3_	*A. cavernosa*	Hachijo-jima Island, Japan	[40]
	-	-	*A. cavernosa*	Shallow water reef in Sanya Bay, Hainan Island, China	[58]
	-	-	*A. cavernosa*	Xisha Islets, South China Sea	[16]
*cis* 10β-Formamidokalihinol A (**90**)	410	C_22_H_35_ClN_2_O_3_	*A. cavernosa*	Shallow water reef in Sanya Bay, Hainan Island, China	[58]
10β-Formamido-5-isocyanatokalihinol A (**91**)	426	C_22_H_35_ClN_2_O_4_	*A. cavernosa*	Hachijo-jima Island, Japan	[40]
	-	-	*A. cavernosa*	Xidao Island, Hainan, China	[48]
10β-Formamido-5β-isothiocyanatokalihinol A (**92**)	442	C_22_H_35_ClN_2_O_3_S	*A. cavernosa*	Hachijo-jima Island, Japan	[40]
	-	-	*A. cavernosa*	Xisha Islets, South China Sea	[16]
	-	-	*A. cavernosa*	Xidao Island, Hainan, China	[48]
Bisformamidokalihinol A (**93**)	428	C_22_H_37_ClN_2_O_4_	*A. cavernosa*	Xidao Island, Hainan, China	[48]
Kalihinol B (**94**)	392	C_22_H_33_ClN_2_O_2_	*Acanthella* sp.	Apra Harbor, Guam, western side of the USA	[22]
	-	-	*Acanthella* sp.	Queen Charlotte Island chain off the coast of British Columbia.	[46]
	-	-	*A. klethra*	Kuchinoerabu Island of the Satsunan Archipelago, Japan	[23]
	-	-	*A. cavernosa*	Seychelles and Desnceufs Islands	[54]
Isokalihinol B (**95**)	392	C_22_H_33_ClN_2_O_2_	*A. klethra*	Kuchinoerabu Island of the Satsunan Archipelago, Japan	[23]
	-	-	*A. cavernosa*	Seychelles and Desnceufs Islands	[54]
	-	-	*A. cavernosa*	Beau Vallon Beach, Mahé, Seychelles	[59]
Kalihinol C (**96**)	328	C_20_H_28_N_2_O_2_	*Acanthella* sp.	Apra Harbor, Guam, western side of the USA	[22]
	-	-	*A. cavernosa*	Seychelles and Desnceufs Islands	[54]
Kalihinol D (**97**)	392	C_22_H_33_ClN_2_O_2_	*Acanthella* sp.	Apra Harbor, Guam, western side of the USA	[22]
	-	-	*A. cavernosa*	Seychelles and Desnceufs Islands	[54]
	-	-	*Acanthella* sp.	Yalong Bay, Hainan, China	[42]
Kalihinol E (**98**)	392	C_22_H_35_ClN_2_O_3_	*Acanthella* sp.	Apra Harbor, Guam, western side of the USA	[22]
	-	-	*A. cavernosa*	Seychelles and Desnceufs Islands	[54]
	-	-	*A. cavernosa*	Hachijo-jima Island, Japan	[40]
	-	-	*A. cavernosa*	Xisha Islets, South China Sea	[16]
	-	-	*A. cavernosa*	South China Sea	[50]
10β-Formamidokalihinol E (**99**)	410	C_22_H_33_ClN_2_O_2_	*A. cavernosa*	Hachijo-jima Island, Japan	[40]
Kalihinol F (**100**)	383	C_23_H_33_N_3_O_2_	*Acanthella* sp.	Apra Harbor, Guam, western side of the USA	[22]
	-	-	*A. caruenosa*	Fiji, South Pacific Ocean	[53]
	-	-	*Acanthella* sp.	Apra Harbor, Guam, western side of the USA	[60]
	-	-	*Acanthella* sp.	Queen Charlotte Island chain off the coast of British Columbia.	[46]
	-	-	*A. klethra*	Kuchinoerabu Island of the Satsunan Archipelago, Japan	[23]
	-	-	*A. cavernosa*	Seychelles and Desnceufs Islands	[54]
	-	-	*A. cavernosa*	Beau Vallon Beach, Mahé, Seychelles	[59]
	-	-	*Acanthella* sp.	Coast of Cape Sada, Ehime Prefecture, Japan	[61]
	-	-	*A. cavernosa*	Dibud, Philippines	[21]
Isokalihinol F (**101**)	383	C_23_H_33_N_3_O_2_	*A. cavernosa*	Fiji, South Pacific Ocean	[53]
	-	-	*A. cavernosa*	Seychelles and Desnceufs Islands	[54]
	-	-	*A. cavernosa*	Beau Vallon Beach, Mahé, Seychelles	[59]
	-	-	*A. cavernosa*	Fiji, South Pacific Ocean	[51]
	-	-	*A. cavernosa*	Heron Island, Great Barrier Reef, Australia	[45]
8-Hydroxy-isokalihinol F (**102**)	399	C_23_H_33_N_3_O_3_	*A. cavernosa*	Heron Island, Great Barrier Reef, Australia	[45]
10-*epi*-Isokalihinol F (**103**)	383	C_23_H_33_N_3_O_2_	*A. cavernosa*	Beau Vallon Beach, Mahé, Seychelles	[59]
*trans* 10-Formamido-kalihinol F (**104**)	401	C_23_H_35_N_3_O_3_	*A. cavernosa*	Dibud, Philippines	[21]
*cis* 10-Formamido-kalihinol F (**105**)	401	C_23_H_35_N_3_O_3_	*A. cavernosa*	Dibud, Philippines	[21]
*trans* 15-Formamido-kalihinol F (**106**)	401	C_23_H_35_N_3_O_3_	*A. cavernosa*	Dibud, Philippines	[21]
*cis* 15-Formamido-kalihinol F (**107**)	401	C_23_H_35_N_3_O_3_	*A. cavernosa*	Dibud, Philippines	[21]
Kalihinol G (**108**)	415	C_23_H_33_N_3_O_2_S	*Acanthella* sp.	Apra Harbor, Guam, western side of the USA	[22]
	-	-	*A. cavernosa*	Seychelles and Desnceufs Islands	[54]
	-	-	*Acanthella* sp.	Coral reef of Ishigaki Island, Okinawa, Japan	[57]
	-	-	*A. cavernosa*	Dibud, Philippines	[21]
10-Isothiocyanatokalihinol G (**109**)	447	C_23_H_33_N_3_O_2_S_2_	*A. cavernosa*	Xisha Islets, South China Sea	[16]
5,10-*bis*-Isothiocyanatokalihinol G (**110**)	479	C_23_H_33_N_3_O_2_S_3_	*Acanthella* sp.	Coral reef of Ishigaki Island, Okinawa, Japan	[57]
Kalihinol H (**111**)	415	C_23_H_33_N_3_O_2_S	*Acanthella* sp.	Apra Harbor, Guam, western side of the USA	[22]
	-	-	*A. cavernosa*	Seychelles and Desnceufs Islands	[54]
	-	-	*Acanthella* sp.	Coral reef of Ishigaki Island, Okinawa, Japan	[57]
Isokalihinol H (**112**)	415	C_23_H_33_N_3_O_2_S	*A. cavernosa*	Beau Vallon Beach, Mahé, Seychelles	[59]
10-*epi*-Isokalihinol H (**113**)	415	C_23_H_33_N_3_O_2_S	*A. cavernosa*	Beau Vallon Beach, Mahé, Seychelles	[59]
Kalihinol I (**114**)	456	C_22_H_33_ClN_2_O_2_S_2_	*A. cavernosa*	Thailand	[18]
	-	-	*A. cavernosa*	Seychelles and Desnceufs Islands	[54]
	-	-	*Acanthella* sp.	Coral reef of Ishigaki Island, Okinawa, Japan	[57]
10-*epi*-Kalihinol I (**115**)	456	C_22_H_33_ClN_2_O_2_S_2_	*Acanthella* sp.	Coral reef of Ishigaki Island, Okinawa, Japan	[57]
	-	-	*A. cavernosa*	Xisha Islets, South China Sea	[16]
	-	-	*A. cavernosa*	South China Sea	[50]
Kalihinol J (**116**)	442	C_22_H_35_ClN_2_O_3_S	*A. cavernosa*	Thailand	[18]
	-	-	*A. cavernosa*	Seychelles and Desnceufs Islands	[54]
	-	-	*A. cavernosa*	Dibud, Philippines	[21]
Kalihinol M (**117**)	442	C_22_H_35_ClN_2_O_3_S	*A. cavernosa*	Xisha Islets, South China Sea	[16]
Kalihinol N (**118**)	442	C_22_H_35_ClN_2_O_3_S	*A. cavernosa*	Xisha Islets, South China Sea	[16]
Kalihinol O (**119**)	424	C_22_H_33_ClN_2_O_2_S	*A. cavernosa*	Xisha Islets, South China Sea	[16]
Kalihinol P (**120**)	424	C_22_H_33_ClN_2_O_2_S	*A. cavernosa*	Xisha Islets, South China Sea	[16]
Kalihinol Q (**121**)	424	C_22_H_33_ClN_2_O_2_S	*A. cavernosa*	Xisha Islets, South China Sea	[16]
Kalihinol R (**122**)	456	C_22_H_33_ClN_2_O_2_S_2_	*A. cavernosa*	Xisha Islets, South China Sea	[16]
Kalihinol S (**123**)	410	C_22_H_35_ClN_2_O_3_	*A. cavernosa*	Xisha Islets, South China Sea	[16]
Kalihinol T (**124**)	424	C_22_H_33_ClN_2_O_2_S	*A. cavernosa*	Xisha Islets, South China Sea	[16]
Kalihinol X (**125**)	424	C_22_H_33_ClN_2_O_2_S	*Acanthella* sp.	Apra Harbor, Guam, western side of the USA	[22]
	-	-	*A. cavernosa*	Fiji, South Pacific Ocean	[53]
	-	-	*A. cavernosa*	Thailand	[18]
	-	-	*A. cavernosa*	Seychelles and Desnceufs Islands	[54]
	-	-	*A. cavernosa*	Dibud, Philippines	[21]
	-	-	*Acanthella* sp.	Yalong Bay, Hainan, China	[42]
10-*epi*-Kalihinol X (**126**)	424	C_22_H_33_ClN_2_O_2_S	*Acanthella* sp.	Yalong Bay, Hainan, China	[42]
	-	-	*A. cavernosa*	Xisha Islets, South China Sea	[16]
	-	-	*A. cavernosa*	South China Sea	[50]
Kalihinol Y (**127**)	365	C_21_H_32_ClNO_2_	*Acanthella* sp.	Apra Harbor, Guam, western side of the USA	[22]
	-	-	*Acanthella caruenosa*	Fiji, South Pacific Ocean	[53]
	-	-	*A. cavernosa*	Thailand	[18]
	-	-	*A. cavernosa*	Seychelles and Desnceufs Islands	[54]
	-	-	*Acanthella* sp.	Coral reef of Ishigaki Island, Okinawa, Japan	[57]
	-	-	*A. cavernosa*	Dibud, Philippines	[21]
Kalihinone Ya (**128**)	367	C_20_H_30_ClNO_3_	*Acanthella* sp.	Apra Harbor, Guam, western side of the USA	[22]
Δ^9^-Kalihinol Y (**129**)	365	C_21_H_32_ClNO_2_	*Acanthella* sp.	Coral reef of Ishigaki Island, Okinawa, Japan	[57]
Kalihinol Z (**130**)	392	C_22_H_33_ClN_2_O_2_	*Acanthella* sp.	Apra Harbor, Guam, western side of the USA	[22]
	-	-	*A. cavernosa*	Fiji, South Pacific Ocean	[53]
	-	-	*A. cavernosa*	Seychelles and Desnceufs Islands	[54]
Kalihiacyloxyamide A (**131**)	468	C_25_H_41_ClN_2_O_4_	*A. cavernosa*	Xisha Island, South China Sea	[62]
Kalihiacyloxyamide B (**132**)	468	C_25_H_41_ClN_2_O_4_	*A. cavernosa*	Xisha Island, South China Sea	[62]
Kalihiacyloxyamide C (**133**)	552	C_30_H_49_ClN_2_O_5_	*A. cavernosa*	Xisha Island, South China Sea	[62]
Kalihiacyloxyamide D (**134**)	552	C_30_H_49_ClN_2_O_5_	*A. cavernosa*	Xisha Island, South China Sea	[62]
Kalihiacyloxyamide E (**135**)	586	C_33_H_47_ClN_2_O_5_	*A. cavernosa*	Xisha Island, South China Sea	[62]
Kalihiacyloxyamide F (**136**)	586	C_33_H_47_ClN_2_O_5_	*A. cavernosa*	Xisha Island, South China Sea	[62]
Kalihiacyloxyamide G (**137**)	518	C_30_H_50_N_2_O_5_	*A. cavernosa*	Xisha Island, South China Sea	[62]
Kalihiacyloxyamide H (**138**)	518	C_30_H_50_N_2_O_5_	*A. cavernosa*	Xisha Island, South China Sea	[62]
Kalihinene					
Kalihinene (**139**)	340	C_22_H_32_N_2_O	*A. klethra*	Kuchinoerabu Island of the Satsunan Archipelago, Japan	[23]
	-	-	*A. cavernosa*	Beau Vallon Beach, Mahé, Seychelles	[59]
	-	-	*A. cavernosa*	Fiji, South Pacific Ocean	[51]
	-	-	*Acanthella* sp.	Coral reef of Ishigaki Island, Okinawa, Japan	[57]
	-	-	*A. cavernosa*	Dibud, Philippines	[21]
	-	-	*Acanthella* sp.	Ximao Sea, Hainan, China	[41]
1-*epi*-Kalihinene (**140**)	340	C_22_H_32_N_2_O	*A. cavernosa*	Beau Vallon Beach, Mahé, Seychelles	[59]
	-	-	*A. cavernosa*	Heron Island, Great Barrier Reef, Australia	[45]
15-Isothiocyanato-l-epi-kalihinene (**141**)	372	C_22_H_32_N_2_OS	*A. cavernosa*	Beau Vallon Beach, Mahé, Seychelles	[59]
1,10-di*epi*-Kalihinene (**142**)	340	C_22_H_32_N_2_O	*A. cavernosa*	Beau Vallon Beach, Mahé, Seychelles	[59]
Kalihinene A (**143**)	340	C_22_H_32_N_2_O	*A. cavernosa*	Seychelles and Desnceufs Islands	[54]
	-	-	*A. cavernosa*	Xisha Islets, South China Sea	[63]
Kalihinene B (**144**)	340	C_22_H_32_N_2_O	*A. cavernosa*	Seychelles and Desnceufs Islands	[54]
Kalihinene E (**145**)	367	C_21_H_34_ClNO_2_	*A. cavernosa*	Xisha Islets, South China Sea	[63]
Kalihinene F (**146**)	331	C_21_H_33_NO_2_	*A. cavernosa*	Xisha Islets, South China Sea	[63]
Kalihinene X (**147**)	367	C_21_H_34_ClNO_2_	*A. cavernosa*	Yakushima Island, southwest of Tokyo	[55,56]
	-	-	*A. cavernosa*	Xisha Islets, South China Sea	[63]
Kalihinene Y (**148**)	367	C_21_H_34_ClNO_2_	*A. cavernosa*	Yakushima Island, southwest of Tokyo	[55,56]
	-	-	*A. cavernosa*	Xisha Islets, South China Sea	[63]
Kalihinene Z (**149**)	367	C_21_H_34_ClNO_2_	*A. cavernosa*	Yakushima Island, southwest of Tokyo	[55,56]
10-Formamidokalihinene (**150**)	358	C_22_H_34_N_2_O_2_	*A. cavernosa*	Yakushima Island, southwest of Tokyo	[55,56]
	-	-	*A. cavernosa*	Fiji, South Pacific Ocean	[51]
	-	-	*A. cavernosa*	Xisha Islets, South China Sea	[63]
15-Formamidokalihinene (**151**)	358	C_22_H_34_N_2_O_2_	*A. cavernosa*	Yakushima Island, southwest of Tokyo	[55]
	-	-	*A. cavernosa*	Fiji, South Pacific Ocean	[51]
	-	-	*A. cavernosa*	Xisha Islets, South China Sea	[63]
10,l5-*bis*-Formamidokalihinene (**152**)	376	C_22_H_36_N_2_O_3_	*A. cavernosa*	Fiji, South Pacific Ocean	[51]
6-Hydroxy-kalihinene (**153**)	356	C_22_H_32_N_2_O_2_	*A. cavernosa*	Fiji, South Pacific Ocean	[51]
	-	-	*Acanthella* sp.	Coral reef of Ishigaki Island, Okinawa, Japan	[57]
6-Hydroxy-15-Formamidokalihinene (**154**)	374	C_22_H_34_N_2_O_3_	*A. cavernosa*	Fiji, South Pacific Ocean	[51]
6-Hydroxy-10-Formamidokalihinene (**155**)	374	C_22_H_34_N_2_O_3_	*A. cavernosa*	Fiji, South Pacific Ocean	[51]
6-Hydroxy-10-Formamido-15-thyocyano-kalihinene (**156**)	406	C_22_H_34_N_2_O_3_S	*A. cavernosa*	Fiji, South Pacific Ocean	[51]
Kalihioxepanes					
Kalihioxepane A (**157**)	365	C_21_H_32_ClNO_2_	*A. cavernosa*	Xisha Island, South China Sea	[64]
Kalihioxepane B (**158**)	365	C_21_H_32_ClNO_2_	*A. cavernosa*	Xisha Island, South China Sea	[64]
Kalihioxepane C (**159**)	383	C_21_H_34_ClNO_3_	*A. cavernosa*	Xisha Island, South China Sea	[64]
Kalihioxepane D (**160**)	383	C_21_H_34_ClNO_3_	*A. cavernosa*	Xisha Island, South China Sea	[64]
Kalihioxepane E (**161**)	383	C_21_H_34_ClNO_3_	*A. cavernosa*	Xisha Island, South China Sea	[64]
Kalihioxepane F (**162**)	365	C_21_H_32_ClNO_2_	*A. cavernosa*	Xisha Island, South China Sea	[64]
Kalihioxepane G (**163**)	365	C_21_H_32_ClNO_2_	*A. cavernosa*	Xisha Island, South China Sea	[64]
Kalihipyran (**164**)	311	C_21_H_29_NO	*A. cavernosa*	Beau Vallon Beach, Mahé, Seychelles	[59]
	-	-	*A. cavernosa*	Heron Island, Great Barrier Reef, Australia	[45]
Kalihipyrans					
Kalihipyran A (**165**)	329	C_21_H_31_NO_2_	*A. cavernosa*	Yakushima Island, southwest of Tokyo	[55]
Kalihipyran B (**166**)	365	C_21_H_32_ClNO_2_	*A. cavernosa*	Yakushima Island, southwest of Tokyo	[55]
Kalihipyran C (**167**)	329	C_21_H_31_NO_2_	*A. cavernosa*	Xisha Islets, South China Sea	[63]
Biflorane diterpenes					
Biflora-4,9,15-triene (**168**)	272	C_20_H_32_	*A. cavernosa*	Yakushima Island, southwest of Tokyo	[55]
	-	-	*A. cavernosa*	Hachijo-jima Island, Japan	[40]
Cavernene A (**169**)	315	C_21_H_33_NO	*A. cavernosa*	Xisha Islets, South China Sea	[63]
Cavernene B (**170**)	317	C_21_H_35_NO	*A. cavernosa*	Xisha Islets, South China Sea	[63]
Cavernene C (**171**)	317	C_21_H_35_NO	*A. cavernosa*	Xisha Islets, South China Sea	[63]
Cavernene D (**172**)	331	C_21_H_33_NO_2_	*A. cavernosa*	Xisha Islets, South China Sea	[63]
Isocyanobifloradiene epoxide A (**173**) 11,12-epoxy-10-isocyano-4,14-bifloradiene (**173**)	313	C_21_H_31_NO	*A. cavernosa*	Heron Island, Great Barrier Reef, Australia	[45]
Isocyanobifloradiene epoxide B (**174**) 11,18-epoxy-10-isocyano-4,14-bifloradiene (**174**)	313	C_21_H_31_NO	*A. cavernosa*	Heron Island, Great Barrier Reef, Australia	[45]

**Table 4:** Alkaloids reported from the genus *Acanthella* (molecular weight and formulae, chemical class, species, and sampling locations).

Compound Name	Mol. Wt.	Mol. Formula	Species	Sampling Locations	Ref.
4-Bromo-1H-pyrrole-2-carboxamide (**175**)	187	C_5_H_5_BrN_2_O	*A. carteri*	Northern coast, Hanish Island Yemen, South Red Sea	[66]
4,5-Dibromo-1H-pyrrole-2-carboxamide (**176**)	265	C_5_H_4_Br_2_N_2_O	*A. carteri*	Northern coast, Hanish Island Yemen, South Red Sea	[66]
Oroidin (**177**)	386	C_11_H_11_Br_2_N_5_O	*A. carteri*	Northern coast, Hanish Island Yemen, South Red Sea	[66]
	-	-	*A. acuta*	Sidi Elghdamssi island, Monastir region, Tunisia	[25]
Axinellizine (**178**)	408	C_11_H_10_Br_2_N_5_NaO	*A. acuta*	Sidi Elghdamssi island, Monastir region, Tunisia	[25]
Hanishin (**179**)	377	C_11_H_12_Br_2_N_2_O_2_	*A. carteri*	Northern coast, Hanish Island Yemen, South Red Sea	[66]
4-(2-Amino-4-oxo-2-imidazolin-5-ylidene)-2-bromo-4,5,6,7-tetrahydropyrrolo [2,3-c] azepin-8-one (**180**)	323	C_11_H_10_BrN_5_O_2_	*A. aurantiaca*	Bay of Naples, southern Italy	[67,68]
4-(2-Amino-4-oxo-2-imidazolin-5-ylidene)-4,5,6,7-tetrahydropyrrolo [2,3-c] azepin-8-one (**181**)	281	C_11_H_12_ClN_5_O_2_	*A. aurantiaca*	Bay of Naples, southern Italy	[67]
Spongiacidin D (**182**)	323	C_11_H_9_BrN_4_O_3_	*A. carteri*	Dalahican Sea, near Ilaya, Lucena, Quezon, Philippines	[17]
3-Bromohymenialdisin (**183**)	402	C_11_H_11_Br_2_N_5_O_2_	*A. carteri*	Dalahican Sea, near Ilaya, Lucena, Quezon, Philippines	[17]
Dihydrospongiacidine (**184**)	404	C_11_H_13_Br_2_N_5_O_2_	*A. carteri*	Dalahican Sea, near Ilaya, Lucena, Quezon, Philippines	[17]
Hymenialdisine (**185**)	323	C_11_H_10_BrN_5_O_2_	*Acanthella* sp.	Yongxing Island, South China Sea	[69]
(-)-Phakellin (**186**)	231	C_11_H_13_N_5_O	*A. costata*	Sykes Reef, Capricorn-Bunker Group Great Barrier Reef, Queensland, Australia	[70]
(-)-Monobromophakellin (**187**)	309	C_11_H_12_BrN_5_O	*A. costata*	Sykes Reef, Capricorn-Bunker Group Great Barrier Reef, Queensland, Australia	[70]
(-)-Dibromophakellin (**188**)	386	C_11_H_11_Br_2_N_5_O	*A. costata*	Sykes Reef, Capricorn-Bunker Group Great Barrier Reef, Queensland, Australia	[70]
Isophakellin (**189**)	231	C_11_H_13_N_5_O	*A. carteri*	Madagascan coast	[71]
Dibromoisophakellin (**190**)	386	C_11_H_11_Br_2_N_5_O	*A. carteri*	Madagascan coast	[71]
Mirabilin G (**191**)	274	C_17_H_28_N_3_	*A. cavernosa*	Southwestern Australia	[24]
Mirabilin K (**192**)	276	C_17_H_30_N_3_	*A. cavernosa*	Southwestern Australia	[24]
Netamine M (**193**)	274	C_17_H_28_N_3_	*A. cavernosa*	Southwestern Australia	[24]
1*H*-Indole-3-carboxylic acid methyl ester (**194**)	175	C_10_H_9_NO_2_	*A. cavernosa*	South China Sea	[50]
1*H*-Indole-3-carboxylic acid (**195**)	161	C_9_H_7_NO_2_	*A. cavernosa*	South China Sea	[50]
Cyclo(L-Phe-L-Leu) (**196**)	260	C_15_H_20_N_2_O_2_	*A. cavernosa*	Coast of Vanua Levu (Fiji island), South Pacific Ocean	[72]
Cyclo(L-Phe-L-Ile) (**197**)	260	C_15_H_20_N_2_O_2_	*A. cavernosa*	Coast of Vanua Levu (Fiji island), South Pacific Ocean	[72]
Cyclo(L-Phe-L-Val) (**198**)	246	C_14_H_18_N_2_O_2_	*A. cavernosa*	Coast of Vanua Levu (Fiji island), South Pacific Ocean	[72]
Cyclo(L-Tyr-L-Ile) (**199**)	276	C_15_H_20_N_2_O_3_	*A. cavernosa*	Coast of Vanua Levu (Fiji island), South Pacific Ocean	[72]
Cyclo(L-Leu-L-Ile) (**200**)	226	C_12_H_22_N_2_O_2_	*A. cavernosa*	Coast of Vanua Levu (Fiji island), South Pacific Ocean	[72]
Cyclo(L-Phe-L-Thr) (**201**)	248	C_13_H_16_N_2_O_3_	*A. cavernosa*	Coast of Vanua Levu (Fiji island), South Pacific Ocean	[72]
Cyclo(L-Phe-L-Tyr) (202)	310	C_18_H_18_N_2_O_3_	*A. cavernosa*	Coast of Vanua Levu (Fiji island), South Pacific Ocean	[72]
Deoxycytidine (**203**)	227	C_9_H_13_N_3_O_4_	*A. cavernosa*	Coast of Vanua Levu (Fiji island), South Pacific Ocean	[72]
Cytosine (**204**)	111	C_4_H_5_N_3_O	*A. cavernosa*	Coast of Vanua Levu (Fiji island), South Pacific Ocean	[72]
Deoxynebularine (**205**)	236	C_10_H_12_N_4_O_3_	*A. cavernosa*	Coast of Vanua Levu (Fiji island), South Pacific Ocean	[72]
Deoxyinosine (**206**)	252	C_10_H_12_N_4_O_4_	*A. cavernosa*	Coast of Vanua Levu (Fiji island), South Pacific Ocean	[72]
Tryptamine (**207**)	160	C_10_H_12_N_2_	*A. cavernosa*	Coast of Vanua Levu (Fiji island), South Pacific Ocean	[72]

**Table 5:** Sterols and other metabolites reported from the genus *Acanthella* (molecular weight and formulae, chemical class, species, and sampling locations).

Compound Name	Mol. Wt.	Mol. Formula	Species	Sampling Locations	Ref.
Steroids					
2β-Hydroxy-4,7-diketo-A-norcholest-5-en-2-oic acid (**208**)	472	C_29_H_44_O_5_	*A. cavernosa*	Inner coral reef, Hainan Island, China	[74]
24*S*-Ethyl-2β-hydroxy-4,7-diketo-A-norcholest-5-en-2-oic acid (**209**)	500	C_31_H_48_O_5_	*A. cavernosa*	Inner coral reef, Hainan Island, China	[74]
2β-Hydroxy-4,7-diketo-24*R*-methyl-A-norcholest-5,22(*E*)-diene-2-oic acid (**210**)	484	C_30_H_44_O_5_	*A. cavernosa*	Inner coral reef, Hainan Island, China	[74]
6α-Hydroxycholest-4-en-3-one (**211**)	400	C_27_H_44_O_2_	*A. cavernosa*	Inner coral reef, Hainan Island, China	[74]
6α-Hydroxyergost-4-en-3-one (**212**)	414	C_28_H_46_O_2_	*A. cavernosa*	Inner coral reef, Hainan Island, China	[74]
Cholest-7,22*E*-dien-3β,5α,6β-triol (**213**)	416	C_27_H_44_O_3_	*A. cavernosa*	Inner coral reef, Hainan Island, China	[74]
24-Norcholest-7,22*E*-dien-3β,5α,6β-triol (**214**)	402	C_26_H_42_O_3_	*A. cavernosa*	Inner coral reef, Hainan Island, China	[74]
5α,8α-Epidioxy-(22*E*,24*R*)-erost-6,22-dien-3β-ol (**215**)	428	C_28_H_44_O_3_	*A. cavernosa*	South China Sea	[50]
Other metabolites					
Isoagelaxanthin A (**216**)	548	C_40_H_52_O	*A. vulgata*	Makura-zaki, Kagoshima, Japan	[75]
Fasciculatin (**217**)	398	C_25_H_34_O_4_	*A. acuta*	Sidi Elghdamssi island, Monastir region, Tunisia	[25]
Hanishenol A (**218**)	424	C_27_H_52_O_3_	*A. carteri*	Northern coast, Hanish Island Yemen, South Red Sea	[76]
Hanishenol B (**219**)	438	C_28_H_54_O_3_	*A. carteri*	Northern coast, Hanish Island Yemen, South Red Sea	[76]
Phalluside 1 (**220**)	739	C_42_H_77_NO_9_	*A. acuta*	Sidi Elghdamssi Island, Monastir region, Tunisia	[25]
Phalluside 2 (**221**)	753	C_43_H_79_NO_9_	*A. acuta*	Sidi Elghdamssi Island, Monastir region, Tunisia	[25]
Phalluside 3 (**222**)	767	C_44_H_81_NO_9_	*A. acuta*	Sidi Elghdamssi Island, Monastir region, Tunisia	[25]
Phenylethylamine (**223**)	121	C_8_H_11_N	*A. cavernosa*	Coast of Vanua Levu (Fiji island), South Pacific Ocean	[72]
2-(4-Hydroxyphenyl)ethylamine (**224**)	137	C_8_H_11_NO	*A. cavernosa*	Coast of Vanua Levu (Fiji island), South Pacific Ocean	[72]
Violacene (**225**)	351	C_10_H_13_BrCl_4_	*Acanthella* sp.	Queen Charlotte Island chain off the coast of British Columbia	[46]
3-Buten-2-one,4-(2,3,6-trimethylphenyl)-3-E (**226**)	188	C_13_H_16_O	*A. cavernosa*	South China Sea	[50]

## Data Availability

Not applicable.

## Abbreviations

A549	Human lung adenocarcinoma epithelial cell line
A2780s	Human ovarian cancer cell line
A2780CP	Human ovarian cancer cell line
ASPC-1	Human pancreatic cancer cell line
CCL2	C–C motif chemokine ligand 2
CD	Circular dichroism
CH_2_Cl_2_	Dichloromethane
CHCl_3_	Chloroform
COX-2	Cyclooxygenase-2
CT-26	Murine colorectal carcinoma cell line
DKPs	Diketopiperazines
DPPH	1,1-diphenyl-2-picrylhydrazyl
EC_50_	Half maximal effective concentration
EtOAc	Ethyl acetate
H69	Chemo-sensitive human small cell lung cancer cell line
H69AR	Chemo-resistant human small cell lung cancer cell line
H1299	Human non-small cell lung carcinoma cell line
HCT-116	Human colon cancer cell line
HeLa	Human cervical epithelioid carcinoma cell line
HPLC	High-performance liquid chromatography
HRESIMS	High-resolution electrospray ionization mass spectroscopy
HT-29	Human colon cancer cell line
IZD	Inhibition zone diameter
K562	Human erythroleukemic cell line
KB	Human oral epidermoid carcinoma cell line
L02	Human liver cell line
LC_50_	Lethal concentration that kills 50%
LD_50_	Half maximal lethal concentration
LD100	Maximal lethal concentration
IR	Infrared
LPS	Lipopolysaccharide
Ls174T	Human colorectal cancer cell line
MDA-MB-231	Human breast cancer cell line
MeOH	Methanol
MRC-5	Human lung fibroblasts
MTT	3-(4,5-dimethylthiazol-2-yl)-2,5-diphenyltetrazolium bromide
n-BuOH	n-butanol
NF-κB	Nuclear factor kappa-light-chain-enhancer of activated B cells
NMR	Nuclear magnetic resonance
P 388	Human leukemia cell line
PC-3	Human prostatic testosterone-independent cell line
PDCD4	Programmed cell death **4**
QGY-7701	Human hepatocellular carcinoma cell line
QM-NMR	Quantum mechanical nuclear magnetic resonance
RP-18	Reversed phase-18
SiO_2_ CC	Silica gel column chromatography
T47D	Human hormone-dependent breast cancer cell line
TDDFT-ECD	Time-dependent density functional theory/electronic circular dichroism
TNF-α	Tumor necrosis factor alpha
TPA	Tetradecanoylphorbol-13-acetate
